# Two entry tunnels in mouse TAAR9 suggest the possibility of multi-entry tunnels in olfactory receptors

**DOI:** 10.1038/s41598-022-06591-z

**Published:** 2022-02-17

**Authors:** ZhengRong Xu, LingNa Guo, XiaoYun Qian, ChenJie Yu, ShengJu Li, ChengWen Zhu, XiaoFeng Ma, Hui Li, GuangJie Zhu, Han Zhou, WenXuan Dai, Qian Li, Xia Gao

**Affiliations:** 1 Department of Otolaryngology Head and Neck Surgery, Jiangsu Provincial Key Medical Discipline (Laboratory), Nanjing Drum Tower Hospital, the Affiliated Hospital of Nanjing University Medical School, Nanjing, 210008 China; 2 Center for Brain Science, Shanghai Children’s Medical Center, School of Medicine, Shanghai Jiao Tong University, Shanghai, 200127 China; 3 Department of Anatomy and Physiology, Ministry of Education-Shanghai Key Laboratory of Children’s Environmental Health in Xinhua Hospital, Shanghai Jiao Tong University School of Medicine, Shanghai, 200025 China; 4 Research Institute of Otolaryngology, Nanjing Drum Tower Hospital, the Affiliated Hospital of Nanjing University Medical School, Nanjing, 210008 China; 5 Shanghai Research Center for Brain Science and Brain-Inspired Intelligence, Shanghai, 201210 China; 6 Key Laboratory for Developmental Genes and Human Disease, Ministry of Education, Jiangsu Province High-Tech Key Laboratory for Bio-Medical Research, Institute of Life Sciences, Southeast University, Nanjing, 210096 China

**Keywords:** Chemical biology, Structural biology

## Abstract

Orthosteric binding sites of olfactory receptors have been well understood for ligand-receptor interactions. However, a lack of explanation for subtle differences in ligand profile of olfactory receptors even with similar orthosteric binding sites promotes more exploration into the entry tunnels of the receptors. An important question regarding entry tunnels is the number of entry tunnels, which was previously believed to be one. Here, we used TAAR9 that recognizes important biogenic amines such as cadaverine, spermine, and spermidine as a model for entry tunnel study. We identified two entry tunnels in TAAR9 and described the residues that form the tunnels. In addition, we found two vestibular binding pockets, each located in one tunnel. We further confirmed the function of two tunnels through site-directed mutagenesis. Our study challenged the existing views regarding the number of entry tunnels in the subfamily of olfactory receptors and demonstrated the possible mechanism how the entry tunnels function in odorant recognition.

## Introduction

The sense of smell mediates perception of external chemical environment in living organisms^1^. Olfactory receptors function in the olfactory subsystems as chemosensors to detect odorants^2,3^. In the main olfactory epithelium (MOE), olfactory receptors mainly consist of two families of class A G protein-coupled receptors (GPCRs): odorant receptors (ORs) and trace amine-associated receptors (TAARs)^4,5^. Each olfactory receptor detects specific odorants and triggers a series of signaling cascades, which in turn transmits the signal to central nervous system and finally generate olfaction^6^. ORs are the largest and highly diverse family of GPCRs in mammals^7^. The OR receptor family can recognize a wide variety of odorants with diverse chemical features, including alcohols, aldehydes, carboxylic acids, esters, ketones, terpenes, and thiols^7,8^. In contrast, TAARs are distantly related to aminergic GPCRs and specifically detect amines^9^.

At molecular level, the transmembrane regions (TMs) and extracellular loops (ECLs) are reported to interact with odorant ligands in olfactory receptors^10,11^. The critical part for odorant recognition is the orthosteric binding pocket, in which odorants bind to specific sites and then activate the receptor^8^. For ORs, orthosteric binding sites include several key residues in the third, fifth, sixth TMs and ECLs^8^. In the case of TAARs, orthosteric binding sites involve a highly conserved aspartic acid in the third TM (Asp^3.32^ or D^3.32^; Ballesteros-Weinstein indexing^12,13^) binding to the amino group of volatile amines through salt bridges and hydrogen bonds^14,15^.

In addition to the orthosteric binding sites, the entry tunnels may also play an important role in determining ligand binding. The entry tunnels reflect the pathway through which the ligands enter the orthosteric binding pockets^16^. Two studies utilizing molecular dynamic analyses on beta-2 adrenergic receptor (β2AR) identified the entry tunnels for ligands to enter the orthosteric binding pockets^17,18^. The entry tunnels are also found in sphingosine 1-phosphate receptor^19^. In olfactory receptors, the location of tunnels in zebrafish TAAR13c for cadaverine entry has been described^20^. Further, when entering the tunnel, cadaverine could bind to an external residue on TM6^20^, which has been defined as vestibule or vestibular binding site in many studies^21^. Odorants can shift back and forth between the vestibular binding pocket and orthosteric binding pocket^22^. Thus, the vestibule could regulate “access control” to the orthosteric sites within the entry tunnels^23^.

A molecular dynamic analysis revealed that the process for ligands switching from vestibular binding pockets to orthosteric binding pockets is very fast and transient^24^. Ligands spend most time in the vestibular binding pocket during the entry process, signifying the importance of vestibular binding pocket to be a functional part of the tunnel. Therefore, the study of vestibular binding pocket is also of great importance to understand the ligand recognition process of receptors. Interestingly, some studies identified one entry tunnel^17–20,25,26^, while others found two^27–32^. In terms of olfactory receptors, the entry tunnels have not been extensively studied, and only one tunnel was reported in TAAR13c^20^.

Taking advantage of the well-studied ligand profile and orthosteric binding mechanism of TAAR^14,15^, we used mouse TAAR9 as a model to study the ligand entry tunnels of olfactory receptors. Previous efforts have identified several ligands for TAAR9, including monoamines such as N-methylpiperidine, triethylamine, N,N-dimethylcyclohexylamine, and polyamines such as 1-(2-aminoethyl)piperidine, cadaverine, spermidine, spermine^9,33^. Firstly, we modeled the structure of TAAR9 and analyzed the intra-receptor interactions which shape the tunnels and the binding pockets. Then, we predicted the entry tunnels of TAAR9 by MOLE2.5 and confirmed them by site-directed mutation experiments. In contrast to previous assumption that there is only one tunnel for ligand entry in TAAR13c, we found two tunnels in TAAR9 with high level of conservation. We also identified one vestibular binding pocket located in each tunnel. At last, we proposed a model to summarize the intra-receptor space where ligands enter, move, and finally bind to the orthosteric binding sites. The discovery of two conservative tunnels suggests the possibility of multiple entry tunnels in the subfamily of olfactory receptors.

## Results

### Intra-receptor interactions in TAAR9

**Figure 1:**
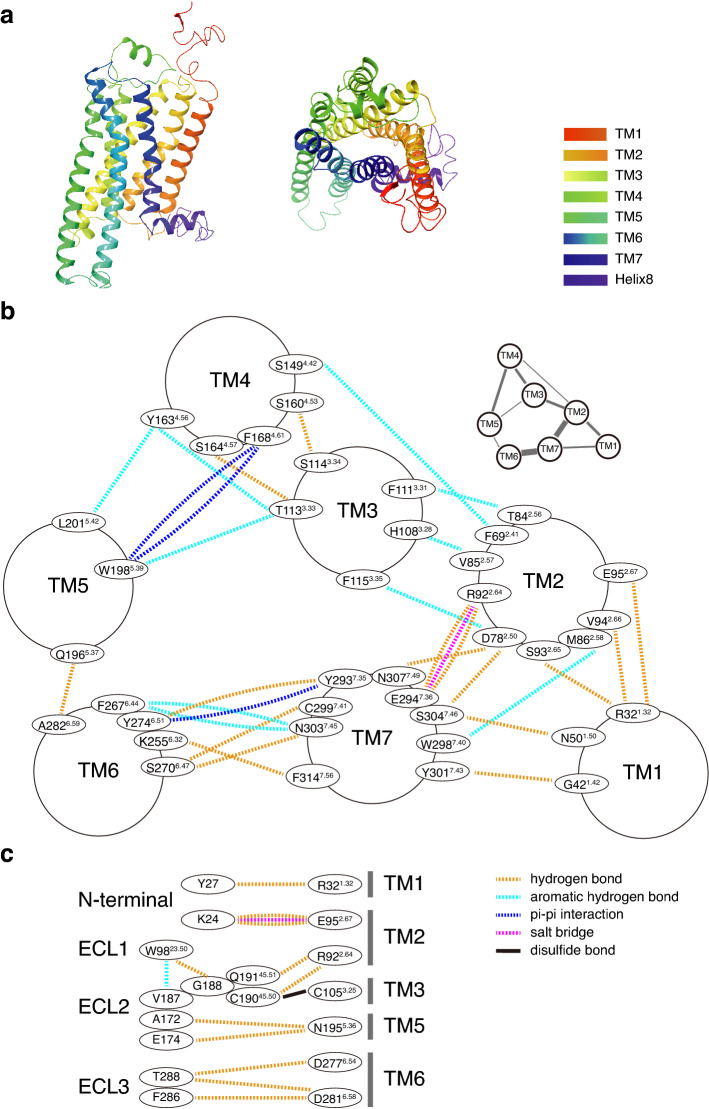
Homology modeling of TAAR9 predicts the intra-receptor interactions. (**a**) Homology modeling of mTAAR9 shows typical 7 transmembrane domains (colored according to residue position). Left: Side view. Right: Extracellular view. (**b**) Detailed inter-helical non-covalent interactions in TAAR9 from extracellular side view. Residue numbers were assigned following numbering scheme in GPCRdb^12^. Dotted lines represent presence of inter-helical interactions. Those interactions are mainly composed of hydrogen bonds (deep yellow) and aromatic-hydrogen bonds (cyan), with a few salt bridges (magenta) and pi-pi interactions (blue). An overview of inter-helical interactions network is shown in upper right, with thicker lines indicating stronger interactions. (**c**) Interactions between TM and N-terminal, TM and ECL, inter-ECL. Besides those interactions described in between TMs, disulfide bond (black lines) is observed between C190^45.50^ and C105^3.25^. Only residues in TM1, 2, 3, 5, 6 show interaction with extracellular domains.

To study the ligand and receptor interaction, we modeled TAAR9 structure using GPCR-I-TASSER, which successfully predicts GPCR structures with high confidence scores^5^. The homology model of TAAR9 was based on crystal structures of eight templates including muscarinic acetylcholine receptor M1, beta-1 adrenergic receptor, beta-2 adrenergic receptor, alpha-2C adrenergic receptor, neuropeptide Y receptor Y1, and muscarinic acetylcholine receptor M4 from different species. The percentage sequence identities of the templates in the threading aligned region are from 23 to 33%. The homology model of TAAR9 meets the criterion of typical class A GPCRs, which have N-terminus, seven transmembrane alpha-helices (TM1-TM7), three extracellular loops (ECL1-ECL3), three intracellular loops (ICL1-ICL3), intracellular amphipathic helix (H8), and C-terminus (Fig. 1a). It also contains a two-turn alpha-helix that packs against ECL2, similar to adenosine A2A receptor^34^.

The intra-receptor interactions between helices shape the space in GPCR, including orthosteric binding pockets, vestibular binding pockets, and entry tunnels^35,36^. By analyzing these interactions and comparing them among different groups of receptors, we can have a better understanding in receptor structural features. So, we firstly summarized these intra-receptor interactions in TAAR9. We observed that most intra-receptor interactions emerge in the outer part of transmembrane regions. 39 residues are found to be involved in the inter-helical interactions including hydrogen bonds, salt bridges, aromatic hydrogen bonds, pi–pi stackings, and pi-cation interactions (Fig. 1b). Most residues interact with only one residue from other transmembrane regions, while some interact with two or more residues. For example, D78^2.50^ connects with three residues from two transmembrane regions, including N307^7.49^, S304^7.46^, and F115^3.35^, forming hydrogen bond, hydrogen bond, and aromatic hydrogen bond, respectively. Among those residues, S304^7.46^ also interacts with N50^1.50^. These complex patterns of interactions together form an interaction network among transmembrane regions (Fig. 1b). TM2 connects four transmembrane domains, including TM1, TM3, TM4, and TM7, suggesting its important role in intra-receptor space shaping. Besides, each of TM3, TM4, TM5, and TM7 connects to three TMs, while each of TM1 and TM6 connects to two TMs (Fig. 1b).

Aside from interactions among transmembrane regions, interactions involving ECLs are also worth noting. The interactions inside ECLs or between ECLs and transmembrane regions may define the receptor conformation and influence the ligand-binding patterns^37^. By summarizing these interactions, we found that TM2 not only interacts with other transmembrane regions, but also interacts with several ECLs (Fig. 1c). For example, we observed hydrogen bonds between E95^2.67^ and K24 in N-terminus, R92^2.64^ and Q191^45.51^, C190^45.50^ in ECL2.

To understand whether those interactions are specific in TAAR9 or conserved in other aminergic receptors, we investigated intra-receptor interactions of another two receptors, mouse TAAR5 and β2AR (PDB entry 3SN6). 31 and 27 interactions are observed in TAAR5 and β2AR, respectively (Supplementary Fig. S1a,b online). Comparing the interactions in the three receptors, we found that only 4 interactions present in all of the three receptors (Supplementary Fig. S1c online), including hydrogen bonds between N^1.50^ and S^7.46^, V^2.57^ and W^3.28^, D^2.50^ and S^7.46^, and disulfide bond between C^45.50^ and C^3.25^. These 4 interactions may be crucial for structural stabilities of aminergic receptors. Among them, the C^3.25^–C^45.50^ disulfide bond has been reported to be necessary for GPCR activation and disruption of it causes a 1000-fold decrease in ligand affinity^38^. It is also worth noting that other 10 interactions are common in TAAR9 and TAAR5 (Supplementary Fig. S1c online). These interactions may be related to TAAR-specific intra-receptor space determination. In contrast, other 4 interactions common in TAAR9 and β2AR, 1 common in TAAR5 and β2AR are found (Supplementary Fig. S1c online), suggesting the differences between TAAR-specific intra-receptor space and other aminergic receptors.

### The predicted ligand binding sites and ligand entry tunnels in TAAR9

**Figure 2:**
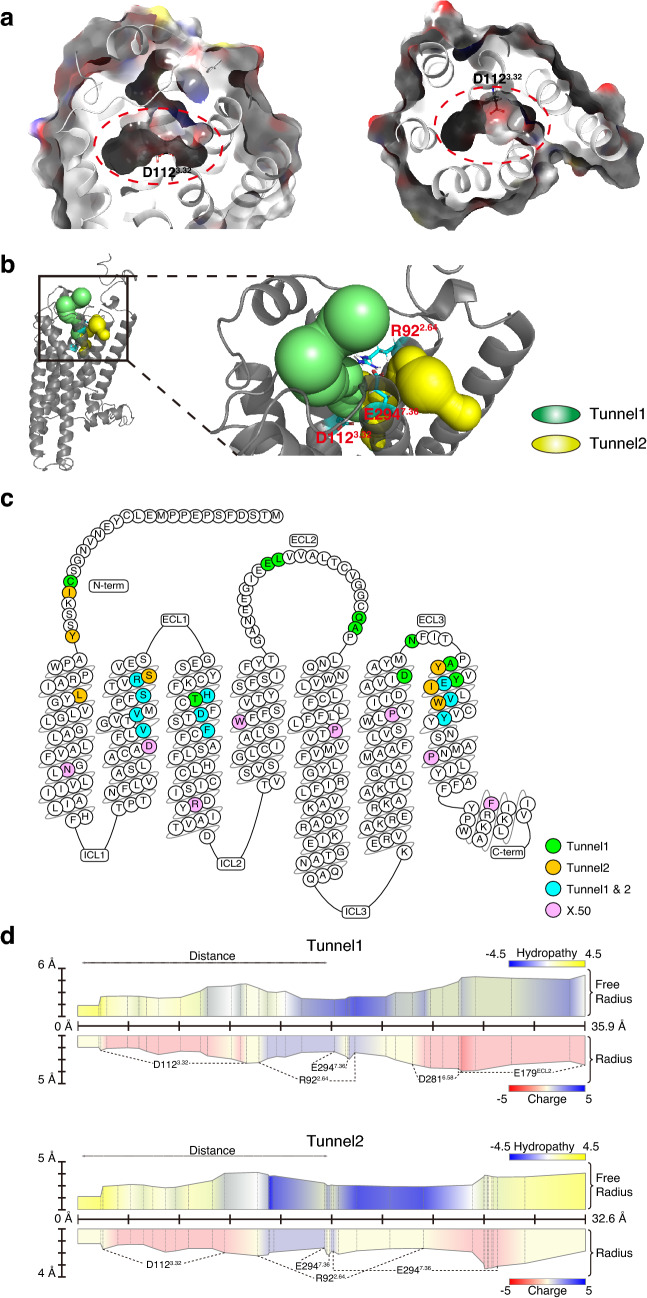
Two predicted tunnels in TAAR9 are considered as pathways for ligand entry. (**a**) Surface (translucent, colored by residue charge) of orthosteric binding pocket (red dotted circle) in side view (left) and extracellular view (right) are located in the center of TMs. D112^3.32^, one of the critical binding sites, is in the center of pocket. (**b**) The location of Tunnel 1 (green) and Tunnel 2 (yellow) in TAAR9. Tunnel 1 and Tunnel 2, which originates from different parts of extracellular regions, converge at the known orthosteric binding site, D112^3.32^. The salt bridge between E294^7.36^ and R92^2.64^ delineates the barrier between Tunnel 1 and Tunnel 2. (**c**) Snake plot for TAAR9 modified from GPCRdb (https://www.gpcrdb.org)^58,59^ demonstrates residues around Tunnel 1 (green), Tunnel 2 (yellow), and both tunnels (cyan). All of the residues are located at the extracellular side of the most conserved residues of each helix (pink), defined as X.50 (X represents TM) on the basis of Ballesteros–Weinstein numbering scheme^13^. (**d**) Properties (length, radius, hydropathy, and charge) of Tunnel 1 (upper) and Tunnel 2 (lower). Both tunnels are hydrophobic (yellow) in internal regions (distance from 0 Å) near the orthosteric binding site, D112^3.32^. Both hydropathy and charge show a distinct pattern between the middle and terminal of the tunnels. Both tunnels are hydrophilic in middle and hydrophobic or neutral in terminals. Distribution of charged residues shows negative charged residues (D112^3.32^, E294^7.36^, D281^6.58^, and E179 in ECL2) in two side and positive charged R92^2.64^ in the middle of both tunnels. Distance, distance (Å) to inside terminal of tunnels. Radius, radius of sphere within tunnel limited by three closest atoms. Free Radius, radius of sphere within tunnel limited by three closest main atoms in order to allow sidechain flexibility. Hydropathy index of amino acid^60^, ranging from the most hydrophilic (Arg = − 4.5) to the most hydrophobic (Ile = 4.5). Charge index of amino acid is summation of the charged residues, with arginine, histidine, and lysine as + 1, and aspartic acid and glutamic acid as − 1.

To acquire the information of tunnels through which ligands enter, we used MOLE2.5, a universal toolkit for analyzing molecular channels and pores, to predict the entry tunnels in TAAR9^39,40^. We obtained 21 tunnels predicted by MOLE2.5 (Supplementary Fig. S2 online) and listed all of the lining residues in these tunnels (Supplementary Table S1 online). Among these, two tunnels were selected for further research because both of them start from the extracellular side and end around the orthosteric binding site, D112^3.32^ (Fig. 2a,b, Supplementary Table S1 online). We named the two tunnels, Tunnel 1 and Tunnel 2 accordingly (Fig. 2b). Tunnel 1 is comprised of residues in TM2, 3, 6, and 7, while Tunnel 2 is comprised of residues in TM1, 2, 3, and 7 (Fig. 2c, Supplementary Table S2 online). Asides from transmembrane residues, Tunnel 1 also contains more extracellular domains including N-terminal, ECL2, and ECL3. In contrast, there are only two residues of N-terminal in Tunnel 2 (Fig. 2c). The two tunnels are similar in the length, with 35.9 Å of Tunnel 1 and 32.6 Å of Tunnel 2 (Fig. 2d, Supplementary Table S3 online). The bottlenecks of both tunnels are also similar at a diameter around 1.3 Å and are located close to the orthosteric binding pocket (Fig. 2d, Supplementary Table S3 online). In addition, minor differences do exist between two tunnels in that the overall charge is -3 in Tunnel 1 while -1 in Tunnel 2, only taking the charged residues into account (Fig. 2d, Supplementary Table S3 online). Even so, the charge distributions of the two tunnels are similar, with negative charge near both terminals and positive change in the middle (Fig. 2d). Other qualities of the two tunnels are similar, except that Tunnel 1 is more hydrophilic than Tunnel 2 (Fig. 2d, Supplementary Table S3 online).

### Both tunnels play important roles in ligand binding

To explore the functions of the two tunnels, we performed site-directed mutagenesis experiments on the key residues in the tunnels. We sought to find the residues that orientate towards the open space of the tunnels and mutate them into larger amino acids with high steric hinderance. We speculated that mutated residues would potentially block the tunnels. We identified 10 specific residues for Tunnel 1 and 7 for Tunnel 2 as shown in Fig. 2c, and carefully analyzed the features of each amino acid (Supplementary Table S4 online). Among them, we chose the residues that might not be involved in functions such as direct ligand-binding and intra-receptor interaction. Besides, the residues in extracellular region that may be very dynamic and the residues near orthosteric binding pocket that may cause instability of orthosteric binding were also excluded. Base on the above considerations, we firstly chose two hydrophobic residues in the middle of two tunnels, A290^7.32^ in Tunnel 1 and L35^1.35^ in Tunnel 2.

**Figure 3:**
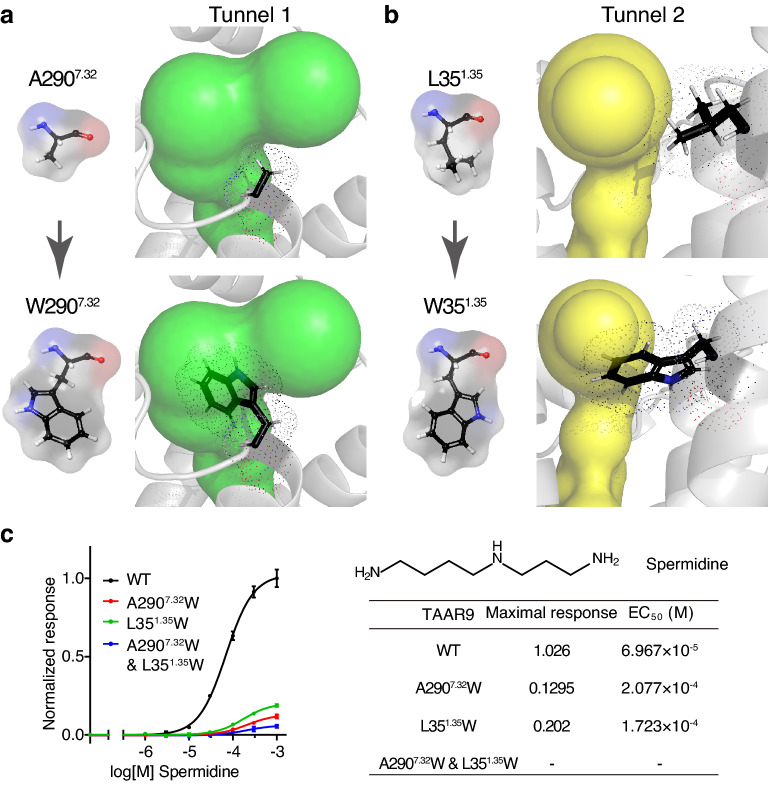
Assessment of the functional role of tunnels. (**a**) Mutation of A290^7.32^ to W290^7.32^ could lead to high steric hinderance in Tunnel 1 (green). (**b**) Mutation of L35^1.35^ to W35^1.35^ could lead to high steric hinderance in Tunnel 2 (yellow). (**c**) Dose-dependent response curves of wild type (WT), A290^7.32^W single mutant, L35^1.35^W single mutant, and A290^7.32^W & L35^1.35^W double mutant TAAR9 to spermidine. The A290^7.32^W or L35^1.35^W single mutant shows much lower maximal response and larger EC_50_. The A290^7.32^W & L35^1.35^W double mutant shows further decrease in maximal response.

Next, we mutated the two residues into tryptophan, one of the largest hydrophobic amino acids^41,42^. In the computational model of TAAR9, we observed that the mutated residue prominently reduces the radius of bottleneck in the corresponding tunnel (Fig. 3a,b). We then applied cell-based assay to examine the responses of mutant TAAR9 to spermidine, the most potent TAAR9 ligand^33^, and compared with those of wild type TAAR9. We observed a 5–10 times decline in maximal response and a 2–3 times increase in EC_50_ of A290^7.32^ W and L35^1.35^ W single mutants (Fig. 3c). Then we obtained a A290^7.32^W & L35^1.35^W double mutant to verify the functional independence of two tunnels. The double mutant barely showed any response to spermidine (Fig. 3c). Taken together, the decreased responses in the A290^7.32^ W and L35^1.35^W single mutants and the near complete loss of response in the A290^7.32^W & L35^1.35^W double mutant strongly suggest that both tunnels contribute to spermidine entry in TAAR9. We also tested the responses of mutant receptors to other TAAR9 ligands, including polyamines (spermine and cadaverine) and monoamines (N-methylpiperidine and triethylamine). The single mutants of tunnel 1 or 2 (A290^7.32^W or L35^1.35^W) decreased the potency and efficacy to both polyamines, while the double mutant (A290^7.32^W & L35^1.35^W) showed no response to both ligands (Supplementary Fig. S3a,b online). Those results are consistent with the entry/docking modeling, suggesting that the two tunnels are also required for entry of spermine and cadaverine in analogy to spermidine. Interestingly, the single mutants (A290^7.32^W or L35^1.35^W) and double mutant (A290^7.32^W & L35^1.35^W) showed decreased receptor response to both monoamines at similar levels (Supplementary Fig. S3c,d online). Those results suggest that the two tunnels are also involved in monoamine entry. However, monoamines seem more tolerant to tunnel mutation than polyamines, possibly due to less positively-charged amino groups of monoamines.

To further validate our findings, we generated single and double mutants of other key sites in Tunnel 1 and Tunnel 2. Those mutants include a group containing single mutants of tunnel 1 or 2 (N285^ECL3^W or L35^1.35^W) and double mutant (N285^ECL3^W & L35^1.35^W), and another group containing single mutants of tunnel 1 or 2 (N285^ECL3^W or I295^7.37^W) and double mutant (N285^ECL3^W & I295^7.37^ W). We observed similar results that single mutants have decreased receptor activity and double mutants have lost receptor activity to spermidine (Supplementary Fig. S3e-g online). There is one exception that I295^7.37^W single mutant has similar response to wild type TAAR9, whereas the double mutant N285^ECL3^W & I295^7.37^W still has no receptor response (Supplementary Fig. S3f,g online). In all, those results strongly suggest that blocking either tunnel could cause an impact on polyamine entry, and blocking both tunnels could result in a dramatic decrease in polyamine entry.

### Residues around two tunnels observed in TAAR9 are largely conserved in all TAARs

Next, we want to investigate if the existence of two functional tunnels can be generalized to a broader range of receptors. Given that biogenic amine receptors are the closest relatives to TAARs^43^, we retrieved amino acid sequences of 50 aminergic receptors in mouse from NCBI, including 15 TAARs, 12 HTRs (serotonin receptors), 5 DRDs (dopamine receptors), 9 ADRs (adrenergic receptors), 5 CHRMs (muscarinic acetylcholine receptors), and 4 HRHs (histamine receptors) (Supplementary Data online). We generated a phylogenetic tree on these 50 receptors (Supplementary Fig. S4a online). The tree clearly distinguished TAARs from other aminergic receptors, suggesting that TAAR family functions differently from other aminergic receptors. We then aligned the 50 sequences and calculated the level of conservation of 27 tunnel-related amino acids in TAAR9 (Supplementary Fig. S4b,c online). We noted that D^3.32^, W^7.40^, and Y^7.43^ in the orthosteric binding sites share high level of conservation among all aminergic receptors. In contrast, most residues identified in the two tunnels of TAAR9 are specific to TAARs, lacking conservation in other aminergic receptors (Supplementary Fig. S4b,c online). Interestingly, we found that TAAR1 has the fewest common residues in the tunnels of TAAR9 among all TAARs, similar to other aminergic receptors (Supplementary Fig. S4d online). A possible explanation could be that TAAR1 functions in the brain, recognizing neurotransmitters like other aminergic receptors rather than olfactory TAARs^9,44^. TAAR5-8 family members have the most common residues with TAAR9 (Supplementary Fig. S4d online), whose ligand profiles are very similar^14^. These phenomena indicate that slight differences of ligand profiles in aminergic receptors could be partially determined by residues along the tunnels.

### Multistep docking suggests that the ligands of TAAR9 linger at D281^6.58^ and E294^7.36^ before progressing to the internal binding pocket

**Figure 4:**
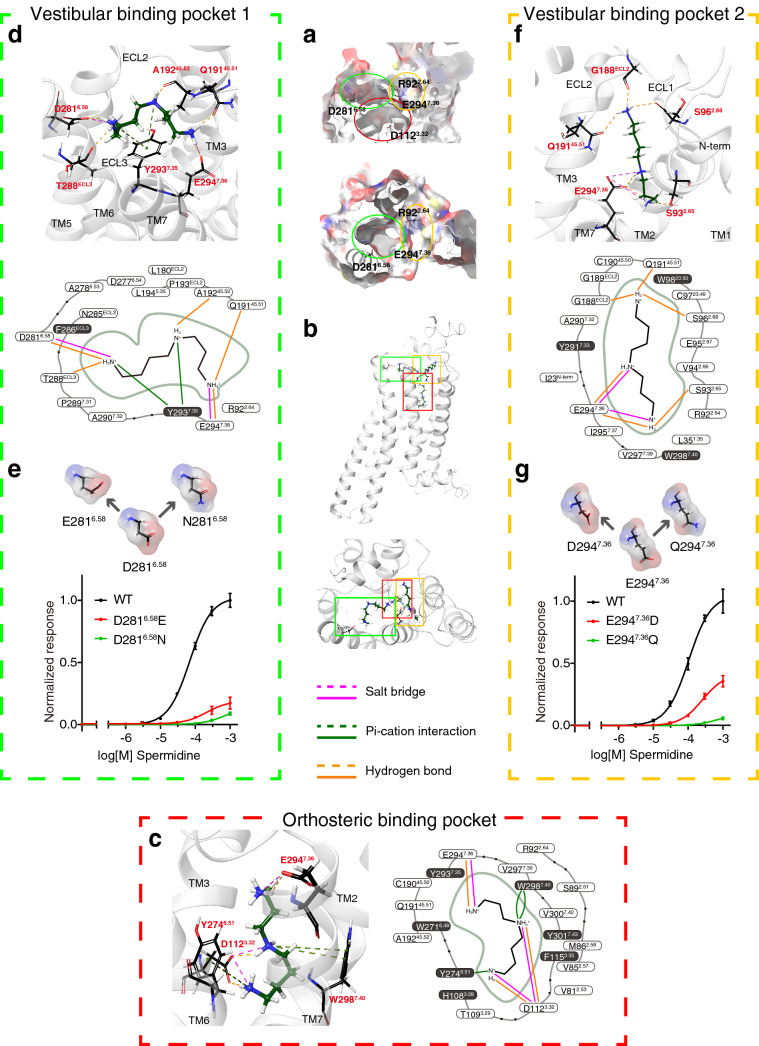
Binding poses of spermidine predicted by multistep induced-fit docking. (**a**) Surface of vestibular binding pocket 1 centered around D281^6.58^, vestibular binding pocket 2 centered around E294^7.36^ and the orthosteric binding pocket centered around D112^3.32^ from side view (upper) and top view (lower). (**b**) Positions of spermidine docked into TAAR9 through multi-step docking. Spermidine can be docked into two vestibular binding pockets and the orthosteric binding pocket described in (**a**). (**c**) Spermidine forms non-covalent bonds with residues in the orthosteric binding pocket and is stabilized by surrounding aromatic rings. The critical binding site, D112^3.32^, forms salt bridges and hydrogen bonds with two amino groups of spermidine. Residues within 5 Å around spermidine were demonstrated. A relatively large proportion of amino acids have the structure of aromatic rings, forming an aromatic cage to stabilize the binding of spermidine. (**d**) Spermidine can be docked into vestibular binding pocket 1, forming salt bridge with D281^6.58^. Side chains of Q191^45.51^ in ECL2, Y293^7.35^, E294^7.36^, and backbones of A192^45.52^ in ECL2, T288 in ECL3 also interact with spermidine. 16 residues within 5 Å range of spermidine are demonstrated. Among them, only 2 have aromatic rings. (**e**) Key residue of vestibular binding pocket 1, D281^6.58^ was mutated to E with altered side chain length but preserved negative charge, and N with similar side chain length but eliminated negative charge. Dose-dependent response curves of WT, D281^6.58^E, and D281^6.58^N mutants show that mutation of D281^6.58^ gives rise to lower receptor responses. (**f**) Spermidine can be docked into vestibular binding pocket 2. Two amino groups of spermidine can interact with E294^7.36^ through salt bridge and hydrogen bond. In addition, side chain of S93^2.65^ can also form hydrogen bond with spermidine. Backbones of three residues including S96^2.68^, G188 in ECL2, and Q191^45.51^ in ECL2 are observed to bind to spermidine in the other terminal. Likewise, residues within 5 Å range of spermidine are demonstrated and only three of them have aromatic rings. (**g**) Mutation of E294^7.36^ to D leads to a significant decrease in receptor activity. Mutation of E294^7.36^ to Q almost eliminates receptor response to spermidine.

To better understand the entry process of TAAR9 ligands, we docked the ligands along the entry tunnels. We divided the tunnels into different segments and each segment was treated individually for docking step thereby identifying optimal docking pose. We used induced-fit docking algorithm integrated in Schrödinger suites to explore the interactions at each step of ligand entry. Surprisingly, spermidine was not distributed equally in each segment of tunnels. Instead, all of the docking results demonstrate that spermidine tends to rest on three specific locations (Fig. 4). In addition to the orthosteric binding pocket which was defined previously, another two locations closer to the extracellular domain were observed in the middle of two tunnels (Fig. 4a,b). According to the previous reports^17^, we assumed that these two pockets were also vestibular binding pockets. Therefore, we named the pocket found in Tunnel 1 as vestibular binding pocket 1, and pocket in Tunnel 2 as vestibular binding pocket 2. To verify the above three pockets predicted in the process of spermidine entry, we also docked another three ligands of TAAR9 including spermine, cadaverine, and 1-(2-aminoethyl)piperdine with the same protocol. We obtained very similar patterns of interactions of TAAR9 with those three ligands, suggesting the common mechanism for ligand entry (Supplementary Figs. S5–S7 online).

We further explored the composition of each pocket and interactions between the ligands and the residues in the pocket. To better locate the pockets, we defined residues within 5 Å of ligands to be the residues constituting the pocket. Furthermore, we summarized the docking results of four TAAR9 ligands and determined the residues as the residues constituting the pocket only when they were conserved in 3 of the 4 ligand docking results. The orthosteric binding pocket is defined by residues in TM2, 3, 6, and 7 (Fig. 4c, Supplementary Figs. S5–S8 online). In the pocket, D112^3.32^ plays a key role in binding spermidine, consistent with previous publications^14^. Additionally, E294^7.36^ can also bind the spermidine through salt bridge and hydrogen bond (Fig. 4c). Aside from salt bridges and hydrogen bonds, residues with aromatic groups bind to spermidine through pi-cation interactions (Fig. 4c). These orthosteric binding sites in TAAR9 were found in the docking results of other three TAAR9 ligands, except that E294^7.36^ is not involved in cadaverine and 1-(2-aminoethyl)piperdine binding (Supplementary Figs. S6, S7 online). Apart from the residues that are directly involved in ligand binding, we identified 15 residues to be the residues constituting the pocket (Supplementary Fig. S8 online). 6 out of 15 residues are amino acids with aromatic groups, including H108^3.28^, F115^3.35^, W271^6.48^, Y274^6.51^, W298^7.40^, and Y301^7.43^. This suggests that the orthosteric binding pocket of TAAR9 is an aromatic cage that encases the ligand firmly for a more stable activation posture.

Vestibular binding pocket 1 in Tunnel 1 opens directly to the surface of cell membrane, and is made of amino acids in ECL2, ECL3, TM6, and TM7. Since ligands in all docking postures connect to D281^6.58^, we considered D281^6.58^ in TM6 to be a critical site in vestibular binding pocket 1. Other sites forming bond with spermidine included E294^7.36^ using salt bridge and hydrogen bond, and Y293^7.35^ using pi-cation interaction (Fig. 4d). In addition, amino acids in extracellular domain also appeared to be involved in spermidine-binding. The backbone carboxyl groups of T288 in ECL3, Q191^45.51^ and A192^45.52^ in ECL2 form hydrogen bonds with spermidine (Fig. 4d). The docking results of spermine, cadaverine and 1-(2-aminoethyl)piperdine also confirmed the critical binding sites including D281^6.58^, E294^7.36^, and Y293^7.35^ (Supplementary Figs. S5–S7 online). Based on our criteria, 11 residues were identified as the residues constituting the pocket (Supplementary Fig. S8 online). In sharp contrast to the orthosteric binding pockets, only 2 of 11 residues contain aromatic groups. The relative loose cage suggests that vestibular binding pocket 1 enables the ligands to move more flexibly. To further confirm the role of D281^6.58^, we performed site-directed mutagenesis and functional assay. We mutated D281^6.58^ into glutamate, which maintains the negative carboxylic acid group but has the longer side chain. We also mutated D281^6.58^ into asparagine, which has a similar steric structure but loses the negative charge. Both D281^6.58^E and D281^6.58^N mutants largely reduced the receptor responses (Fig. 4e, Supplementary Fig. S9a online), confirming the importance of D281^6.58^ in ligand recognition.

Vestibular binding pocket 2 is constituted by amino acids in ECL1, ECL2, TM2, and TM7. In this pocket, E294^7.36^ that exists in all docking posture is regarded as the critical binding residue. Other residues include S93^2.65^ and S96^2.68^ in TM2, G188 and Q191^45.51^ in ECL2 that form hydrogen bonds with spermidine (Fig. 4f). Similar binding patterns were observed in spermine, cadaverine, and 1-(2-aminoethyl)piperidine (Supplementary Figs. S5–S7 online). Based on our criteria, 18 residues were identified as the residues constituting the pocket (Supplementary Fig. S8 online). Similar to vestibular binding pocket 1, only 3 of them contain aromatic rings, suggesting that the two vestibular binding pockets have a common function as a transitional step in ligand entry. Similarly, we mutated E294^7.36^ into aspartate with preserved negative charge and shorter side chain, and glutamine with the same length of side chain and eliminated negative charge. E294^7.36^D mutant showed decreased response, while E294^7.36^Q mutant showed much lower response, suggesting the importance of E294^7.36^ in ligand recognition (Fig. 4g, Supplementary Fig. S9b online).

To exclude the possibility that reduced receptor responses are resulted from reduction in surface expression levels, we performed fluorescence-activated cell sorting analysis (FACS). All of the mutants tested showed comparable surface expression levels with wild type TAAR9 (Supplementary Fig. S10 online). Therefore, we think that receptor trafficking is not dramatically altered by receptor mutation. However, we could not rule out other possibilities such as receptor coupling changes caused by mutagenesis.

### Negatively charged glutamic acids in ECL2 of TAAR9 may also participate in ligand recognition

Since our predicted Tunnel 1 also touches some ECL2 residues, we extended our studies beyond tunnels and vestibular binding pockets to the function of extracellular domains. ECL2, located between TM4 and TM5, is the longest extracellular loop of most GPCRs. Previous molecular dynamic analysis revealed that ECL2s in β2AR and muscarinic acetylcholine receptors function to recruit extracellular ligands^17,45^. It has also been reported that ECL2 takes part in allosteric binding^37^, and mutation of residues in ECL2 could affect the ligand profile^46^. In TAAR9, we found that spermidine can be docked into a pose that binds to two negatively charged residues, E178 and E179 in ECL2 (Supplementary Fig. S11a online). Both residues are located in a short alpha-helix in ECL2 (Supplementary Fig. S11a online), and only E179 is considered a part of tunnel by prediction (Fig. 2c). To confirm the function of these two amino acids, we generated single or double mutants and examined their responses to spermidine. In both E178A and E179A single mutants, no significant differences in EC_50_ were found compared to wild type TAAR9 (Supplementary Fig. S11b online). By contrast, a 3.6-fold increase in EC_50_ was found in E178 & E179 double mutant. It is possible that E178 and E179 are two key residues to recruit TAAR9 ligand. Loss of one residue can be compensated by the other due to the relative short distance between them (Supplementary Fig. S11c online). Besides, we noted another two sequential glutamate residues in ECL2, E174 and E175. We sought to explore if they have similar functions with E178 and E179. However, neither single nor double mutants in E174 and E175 showed significant changes in EC_50_ (Supplementary Fig. S11b online), suggesting that E174 and E175 are not directly involved in ligand recruitment or binding.

## Discussion

In our study on ligand entry in TAAR9, we identified two potential tunnels that connect the external surface of the receptor to the internal orthosteric binding pocket. To verify the function of two tunnels, we mutated the residues along the tunnels to generate a higher steric hinderance and found a significant decrease in the receptor response to TAAR9 ligands. In contrast to previous study on zebrafish TAAR13c that claimed only one functional tunnel^20^, we confirmed that both tunnels contribute to ligand entry in TAAR9. Moreover, the two tunnels seem to have a higher level of conservation in the TAAR family.

A possible explanation for the fact of one tunnel in TAAR13c and two tunnels in TAAR9 could be the difference of a critical residue, 7.36 in the receptors. TAAR13c has D^7.36^, while TAAR9 has E^7.36^, which forms a salt bridge with R^2.64^ delineating the boundaries of the two tunnels. Therefore, a change in this site may give rise to the differences of tunnel space. Moreover, the tunnels we predicted in TAAR9 are slightly different from that in TAAR13c. For example, both of the two tunnels pass R^2.64^ in TAAR9, while the tunnel in TAAR13c does not pass R^2.64^. Besides, TAAR13c belongs to a teleost-specific TAAR subfamily^15^. The distinct characteristics in bony fish TAARs and other species-specific TAAR may require further investigation.

Inside the two entry tunnels, we also identified two vestibular binding pockets. The two vestibular binding pockets are characterized by functional negative-charged residues, whose roles are confirmed by site-directed mutation experiments. There are fewer surrounding aromatic rings in the vestibular binding pockets than in the orthosteric binding pocket. The negatively charged residues in the vestibular binding pockets may function to attract the ligands from extracellular regions to the pockets. After the ligands enter one of the vestibular pockets, the pocket environment with fewer aromatic rings makes the ligand-receptor posture less stable, facilitating their entry into the orthosteric binding pocket. Similar vestibular binding pocket patterns were described in previous studies. D^6.58^ was proved to be a vestibular binding site in zebrafish TAAR13c^20^. H^6.58^ was reported as a component of vestibular binding sites highly conserved in class I OR^22^. Another site, 7.36, also affects ligand binding in other aminergic receptors^47,48^ as well as a bitter taste receptor, TAS2R46^23^. Therefore, vestibular binding pockets centered around these two residues may exist in a wide range of GPCRs.

**Figure 5:**
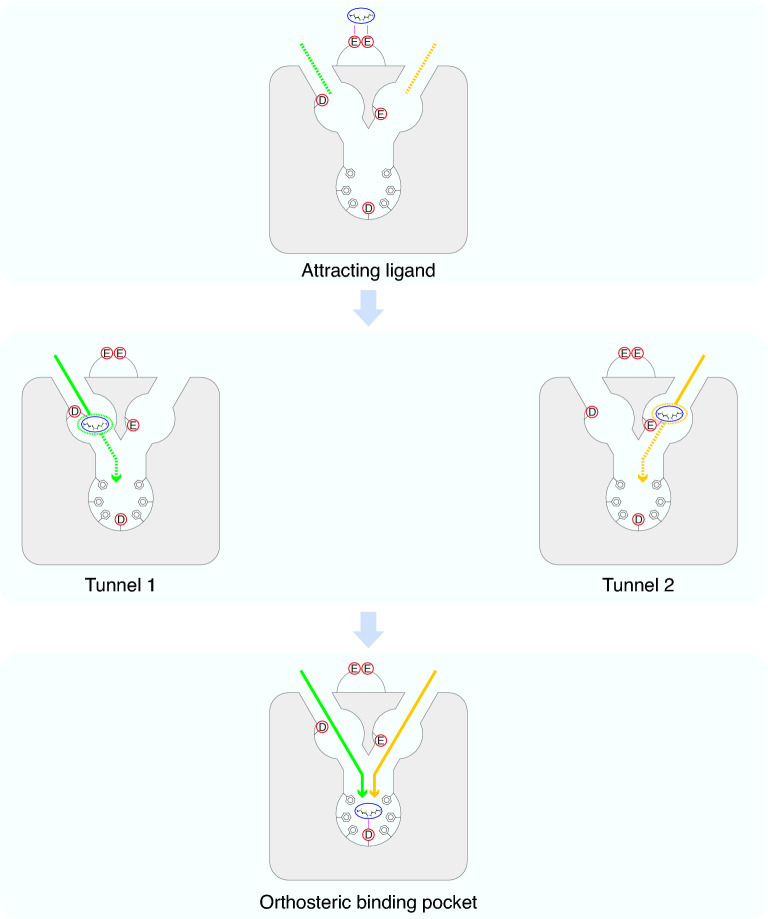
Schematic illustration of ligand-binding process of TAAR9. Firstly, the negative-charged glutamates in ECL2 attract the positive-charged amine to approach the opening of tunnels in the receptor (top). Then the amine ligand can enter either vestibular binding pocket 1 through Tunnel 1 (green) interacting with D^6.58^ (middle left), or vestibular binding pocket 2 through Tunnel 2 (yellow) interacting with E^7.36^ (middle right). Both tunnels lead to the orthosteric binding pocket, where the amine ligand can bind firmly with D^3.32^ and gets stabilized with surrounding residues having aromatic rings (bottom).

In summary, we described a possible route for odorants dissolved in olfactory mucus to activate TAAR. Firstly, amines that are positively charged under physiological conditions are recruited by the negatively charged glutamates in ECL2. As amines approach closer to the receptor, they can enter either one of the two tunnels. In Tunnel 1, D^6.58^ is mainly responsible for ligands to enter vestibular binding pocket 1. In Tunnel 2, E^7.36^ is the critical residue for ligands to enter vestibular binding pocket 2. Finally, the odorants enter the orthosteric binding pocket through any of the two tunnels and bind to D^3.32^, which is further stabilized by surrounding aromatic residues in the orthosteric binding pocket (Fig. 5). In all, our study on entry tunnels and vestibular binding pockets provides a novel insight into ligand recognition pattern in olfactory receptors.

## Methods

### Computational analysis of tunnels and binding pockets

Homology models of TAAR9 and TAAR5 were generated by an online structure prediction tool, GPCR-I-TASSER (https://zhanglab.ccmb.med.umich.edu/GPCR-I-TASSER/)^49^. This method has been considered as one of the most efficient modeling methods for GPCR, and was widely used by many researches^50–52^. The website provided several possible TAAR9 structures, among which the model with highest confidence score was chosen for further research. The homology model of TAAR9 was based on crystal structures of eight templates (Protein Data Bank [PDB] Entry 6oijR, 4amjA, 6kuwA, 5zbh, 3d4s, 5jqhA, 6kp6A, and 3sn6R). The intra-receptor interactions were visualized in Maestro of Schrödinger suites (released 2020-1). The tunnels in TAAR9 were predicted in MOLE2.5 online (https://mole.upol.cz/)^40^ and visualized in PyMOL v2.4.1. Docking of ligands were performed using Maestro of Schrödinger suites. TAAR9 model was prepared for docking by Protein Preparation Wizard module^53^. Protonation and charge states were generated in physiological environment (pH 7.0 ± 2.0). The model underwent further preparation procedures including hydrogen bond optimization and restrained minimization. The structures of ligands were retrieved from Pubchem (https://pubchem.ncbi.nlm.nih.gov) and prepared by LigPrep module to adapt to the physiological environment. Receptor-ligand docking within two tunnels were performed using induced-fit docking module^54^. The boxes limiting ranges of ligands were defined by residues in each segment predicted by MOLE2.5. Top ten poses of ligand-receptor interactions were generated for further analysis and final poses were chosen according to docking score. The mutant structures were generated by wizard mutagenesis protein module integrated in PyMOL. All sequences used to construct the phylogenetic tree were retrieved from NCBI (https://www.ncbi.nlm.nih.gov). Multi-sequence alignment was performed in MAFFT v7.313^55^. Maximum likelihood phylogenetic tree was generated using IQtree v1.6.8^56^. Visualization and modification of the tree were performed in Figtree v1.4.4 (http://tree.bio.ed.ac.uk/software/figtree/).

### Site-directed mutagenesis

Mouse TAAR9 gene was cloned from mouse genomic DNA to modified pcDNA3.1(-) vector with 20-amino acid Rho tag (MNGTEGPNFYVPFSNKTGVV) in the N-terminus. Site-directed mutagenesis of mouse TAAR9 was performed by PCR-based site-directed mutagenesis following the protocol of TIANGEN fast site-directed mutagenesis kit (Tiangen Biotech, Beijing, China). The DpnI-incubated products were transferred into DMT competent cells (Tiangen Biotech, Beijing, China). All mutants were verified by Sanger sequencing.

### Dual-Luciferase reporter assay

TAAR9 and mutants were transiently expressed in Hana3A cell line derived from HEK293T cell line^57^. Cells were grown in poly-D-lysine coated 96-well plates to a density of 1 × 10^4^ cells per well and loaded at 37 °C with 5% CO_2_. After 24 h, 50 ng receptor plasmids with 10 ng pCRE-LUC, 10 ng pSV40-RL, 10 ng mRTP1S were transfected using Lipofectamine 2000 (Invitrogen) and remained incubation for 18 h. Then the media was replaced by 50 μL CD293 media (GIBCO) with 1% glutamine and incubated for 30 min. Ligand compounds (Sigma-Aldrich) diluted in CD293 media at proper concentrations were transferred to each well and cultured for 4 h. Dual-Glo luciferase reagent (Promega) was added to each well and the plate was rotated for 10 min. The firefly luciferase activity (Luc) and renilla luciferase activity (RL) were measured using BioTek microplate reader. Luc/RL values were calculated by firefly luciferase divided by renilla luciferase of each well. Afterwards, values were subtracted by the average values of wells without ligand stimulation, and were then divided by the maximal values from wild type TAAR9 stimulated by ligand to obtain the normalized data. Note that it is regarded as no responses if the Luc/RL values of maximal responses are less than two-fold of the Luc/RL values of wells without ligand.

### FACS analysis

The density of 3 × 10^5^ Hana3A cells per well were incubated in 6-well plates at 37 °C with 5% CO_2_. After 24 h, cells were transfected with 2 μg receptor plasmids (wild type or mutant TAAR9), 1 μg mRTP1S, and 0.2 μg pEGFP and incubated for 18 h. After dissociation using CellstripperTM (Corning), cells in each well were transferred into 2 mL tubes with 100 μL staining buffer (5% BSA and 1% NaN_3_ in PBS) containing anti-Rho tag primary antibody (MABN15, Millipore, 1:100) and incubated at 4 °C for 45 min. Cells were washed twice by adding 2 mL staining buffer and centrifuged at 200×*g* at 4 °C for 3 min. Subsequently, 100 μL staining buffer containing phycoerythrin-conjugated donkey anti-mouse secondary antibody (Jackson ImmunoResearch, 1:100) was applied at 4 °C for 30 min. Cells were then washed twice by adding 2 mL staining buffer and centrifuged at 200×*g* at 4 °C for 3 min. The fluorescence was measured using flow cytometry (BD LSRFortessaTM X-20, Becton). The ratios of Rho tag and EGFP double positive cells to the EGFP single positive cells were considered as the level of cell surface expression.

## Supplementary Information

Supplementary Information.

## References

[1] Bear DM, Lassance JM, Hoekstra HE, Datta SR (2016). The evolving neural and genetic architecture of vertebrate olfaction. Curr. Biol..

[2] Bjarnadottir TK (2006). Comprehensive repertoire and phylogenetic analysis of the G protein-coupled receptors in human and mouse. Genomics.

[3] Spehr M, Munger SD (2009). Olfactory receptors: G protein-coupled receptors and beyond. J. Neurochem..

[4] Liberles SD, Buck LB (2006). A second class of chemosensory receptors in the olfactory epithelium. Nature.

[5] Buck L, Axel R (1991). A novel multigene family may encode odorant receptors: A molecular basis for odor recognition. Cell.

[6] Silva Teixeira CS, Cerqueira NM, Silva Ferreira AC (2016). Unravelling the olfactory sense: From the gene to odor perception. Chem. Senses.

[7] Ikegami K (2020). Structural instability and divergence from conserved residues underlie intracellular retention of mammalian odorant receptors. Proc. Natl. Acad. Sci. U S A.

[8] Block E (2018). Molecular basis of mammalian odor discrimination: A status report. J. Agric. Food Chem..

[9] Xu Z, Li Q (2020). TAAR agonists. Cell Mol. Neurobiol..

[10] Floriano WB, Vaidehi N, Goddard WA, Singer MS, Shepherd GM (2000). Molecular mechanisms underlying differential odor responses of a mouse olfactory receptor. Proc. Natl. Acad. Sci. U S A.

[11] Man O, Gilad Y, Lancet D (2004). Prediction of the odorant binding site of olfactory receptor proteins by human-mouse comparisons. Protein Sci..

[12] Isberg V (2015). Generic GPCR residue numbers: Aligning topology maps while minding the gaps. Trends Pharmacol. Sci..

[13] Ballesteros JA, Weinstein H, Sealfon SC (1995). Methods in Neuroscience.

[14] Ferrero DM (2012). Agonists for 13 trace amine-associated receptors provide insight into the molecular basis of odor selectivity. ACS Chem. Biol..

[15] Li Q (2015). Non-classical amine recognition evolved in a large clade of olfactory receptors. Elife.

[16] Piechnick R (2012). Effect of channel mutations on the uptake and release of the retinal ligand in opsin. Proc. Natl. Acad. Sci. U S A.

[17] Dror RO (2011). Pathway and mechanism of drug binding to G-protein-coupled receptors. Proc. Natl. Acad. Sci. U S A.

[18] Wang T, Duan Y (2009). Ligand entry and exit pathways in the beta2-adrenergic receptor. J. Mol. Biol..

[19] Hanson MA (2012). Crystal structure of a lipid G protein-coupled receptor. Science.

[20] Sharma K, Balfanz S, Baumann A, Korsching S (2018). Full rescue of an inactive olfactory receptor mutant by elimination of an allosteric ligand-gating site. Sci. Rep..

[21] Bock A (2012). The allosteric vestibule of a seven transmembrane helical receptor controls G-protein coupling. Nat. Commun..

[22] Bushdid C (2019). Mammalian class I odorant receptors exhibit a conserved vestibular-binding pocket. Cell Mol. Life Sci..

[23] Sandal M (2015). Evidence for a transient additional ligand binding site in the TAS2R46 bitter taste receptor. J. Chem. Theory Comput..

[24] Strasser A, Wittmann HJ, Seifert R (2017). Binding kinetics and pathways of ligands to GPCRs. Trends Pharmacol. Sci..

[25] Stanley N, Pardo L, Fabritiis GD (2016). The pathway of ligand entry from the membrane bilayer to a lipid G protein-coupled receptor. Sci. Rep..

[26] Selent J, Sanz F, Pastor M, De Fabritiis G (2010). Induced effects of sodium ions on dopaminergic G-protein coupled receptors. PLoS Comput. Biol..

[27] Gonzalez A, Perez-Acle T, Pardo L, Deupi X (2011). Molecular basis of ligand dissociation in beta-adrenergic receptors. PLoS ONE.

[28] Mustafi D, Palczewski K (2009). Topology of class A G protein-coupled receptors: Insights gained from crystal structures of rhodopsins, adrenergic and adenosine receptors. Mol. Pharmacol..

[29] Schadel SA (2003). Ligand channeling within a G-protein-coupled receptor. The entry and exit of retinals in native opsin. J. Biol. Chem..

[30] Hildebrand PW (2009). A ligand channel through the G protein coupled receptor opsin. PLoS ONE.

[31] Guixa-Gonzalez R (2017). Membrane cholesterol access into a G-protein-coupled receptor. Nat. Commun..

[32] Wittmann HJ, Strasser A (2015). Binding pathway of histamine to the hH4R, observed by unconstrained molecular dynamics. Bioorg. Med. Chem. Lett..

[33] Saraiva LR (2016). Combinatorial effects of odorants on mouse behavior. Proc. Natl. Acad. Sci. U S A.

[34] Venkatakrishnan AJ (2013). Molecular signatures of G-protein-coupled receptors. Nature.

[35] Rosenkilde MM, Benned-Jensen T, Frimurer TM, Schwartz TW (2010). The minor binding pocket: A major player in 7TM receptor activation. Trends Pharmacol. Sci..

[36] Heifetz A (2020). Characterizing interhelical interactions of G-protein coupled receptors with the fragment molecular orbital method. J. Chem. Theory Comput..

[37] Wheatley M (2012). Lifting the lid on GPCRs: The role of extracellular loops. Br. J. Pharmacol..

[38] Fraser CM (1989). Site-directed mutagenesis of beta-adrenergic receptors. Identification of conserved cysteine residues that independently affect ligand binding and receptor activation. J. Biol. Chem..

[39] Sehnal D (2013). MOLE 2.0: Advanced approach for analysis of biomacromolecular channels. J. Cheminform..

[40] Berka K (2012). MOLEonline 2.0: Interactive web-based analysis of biomacromolecular channels. Nucleic Acids Res..

[41] Taylor IR (2020). Tryptophan scanning mutagenesis as a way to mimic the compound-bound state and probe the selectivity of allosteric inhibitors in cells. Chem. Sci..

[42] Rasmussen T (2015). Properties of the mechanosensitive channel MscS pore revealed by tryptophan scanning mutagenesis. Biochemistry.

[43] Pandy-Szekeres G (2018). GPCRdb in 2018: Adding GPCR structure models and ligands. Nucleic Acids Res..

[44] Gainetdinov RR, Hoener MC, Berry MD (2018). Trace amines and their receptors. Pharmacol. Rev..

[45] Kruse AC (2012). Structure and dynamics of the M3 muscarinic acetylcholine receptor. Nature.

[46] Shi L, Javitch JA (2002). The binding site of aminergic G protein-coupled receptors: The transmembrane segments and second extracellular loop. Annu. Rev. Pharmacol. Toxicol..

[47] Vass M (2019). Aminergic GPCR-ligand interactions: A chemical and structural map of receptor mutation data. J. Med. Chem..

[48] Jia L (2021). Convergent olfactory trace amine-associated receptors detect biogenic polyamines with distinct motifs via a conserved binding site. J. Biol. Chem..

[49] Zhang J, Yang J, Jang R, Zhang Y (2015). GPCR-I-TASSER: A hybrid approach to G protein-coupled receptor structure modeling and the application to the human genome. Structure.

[50] White AD (2019). Ca(2+) allostery in PTH-receptor signaling. Proc. Natl. Acad. Sci. U S A.

[51] Jorgensen AS (2021). Biased action of the CXCR4-targeting drug plerixafor is essential for its superior hematopoietic stem cell mobilization. Commun. Biol..

[52] Rowe JB, Kapolka NJ, Taghon GJ, Morgan WM, Isom DG (2021). The evolution and mechanism of GPCR proton sensing. J. Biol. Chem..

[53] Sastry GM, Adzhigirey M, Day T, Annabhimoju R, Sherman W (2013). Protein and ligand preparation: Parameters, protocols, and influence on virtual screening enrichments. J. Comput. Aided Mol. Des..

[54] Sherman W, Beard HS, Farid R (2006). Use of an induced fit receptor structure in virtual screening. Chem. Biol. Drug Des..

[55] Katoh K, Standley DM (2013). MAFFT multiple sequence alignment software version 7: Improvements in performance and usability. Mol. Biol. Evol..

[56] Nguyen LT, Schmidt HA, von Haeseler A, Minh BQ (2015). IQ-TREE: A fast and effective stochastic algorithm for estimating maximum-likelihood phylogenies. Mol. Biol. Evol..

[57] Zhuang H, Matsunami H (2008). Evaluating cell-surface expression and measuring activation of mammalian odorant receptors in heterologous cells. Nat. Protoc..

[58] Isberg V (2016). GPCRdb: an information system for G protein-coupled receptors. Nucleic Acids Res.

[59] Kooistra AJ (2021). GPCRdb in 2021: Integrating GPCR sequence, structure and function. Nucleic Acids Res..

[60] Kyte J, Doolittle RF (1982). A simple method for displaying the hydropathic character of a protein. J. Mol. Biol..

