# Supplementary material for: Two entry tunnels in mouse TAAR9 suggest the possibility of multi-entry tunnels in olfactory receptors
Source: Sci Rep. 2022 Feb 17;12:2691. doi: 10.1038/s41598-022-06591-z (PMC8854740; doi:10.1038/s41598-022-06591-z)
Supplement: Supplementary file 1 — Supplementary Information. [file 41598_2022_6591_MOESM1_ESM.pdf]

Supplementary Information

Two entry tunnels in mouse TAAR9 suggest the possibility of multi-entry tunnels in olfactory receptors

Authors: ZhengRong Xu[1,2,3,4,‡], LingNa Guo [2,3,6,‡], XiaoYun Qian[1,4,‡], ChenJie Yu[1,4,‡], ShengJu Li [2,3], ChengWen Zhu[1,4], XiaoFeng Ma[1,4], Hui Li[1,4], GuangJie Zhu[1,4], Han Zhou[1,4], WenXuan Dai[2,3,*], Qian Li[2,3,5,*] & Xia Gao[1,4,*]

Correspondence to: xiagaogao@hotmail.com; liqian@shsmu.edu.cn; 518710910026@shsmu.edu.cn

This PDF file includes:

Supplementary Figure S1. TAAR9 has a higher level of conservation in intra-receptor interaction with TAAR5 than with β2AR.

Supplementary Figure S2. 21 tunnels of TAAR9 predicted by MOLE2.5.

Supplementary Figure S3. Responses of additional TAAR9 mutants to other TAAR9 ligands.

Supplementary Figure S4. Conservation of residues along two tunnels of TAAR9 in aminergic receptors.

Supplementary Figure S5. Binding poses of spermine predicted by multistep induced-fit docking.

Supplementary Figure S6. Binding poses of cadaverine predicted by multistep induced-fit docking.

Supplementary Figure S7. Binding poses of 1-(2-aminoethyl)piperidine predicted by multistep induced-fit docking.

Supplementary Figure S8. Most of the residues within 5 Å of the orthosteric binding pocket of four ligands are in common.

Supplementary Figure S9. Mutations of residues in vestibular binding pockets lead to changes of ligand recognition.

Supplementary Figure S10. Analysis of cell-surface expression by FACS.

Supplementary Figure S11. Elimination of negative charge of some glutamate residues in ECL2 of TAAR9 decreases its ligand binding efficacy.

Table S1. Residues in the 21 tunnels predicted by MOLE2.5.

Table S2. Residues around two tunnels.

Table S3. Values of properties of two tunnels offered by MOLE website.

Table S4. Locations and features of 17 residues specific to each tunnel.

Supplementary Data. Amino acid sequences of 50 mouse aminergic receptors.

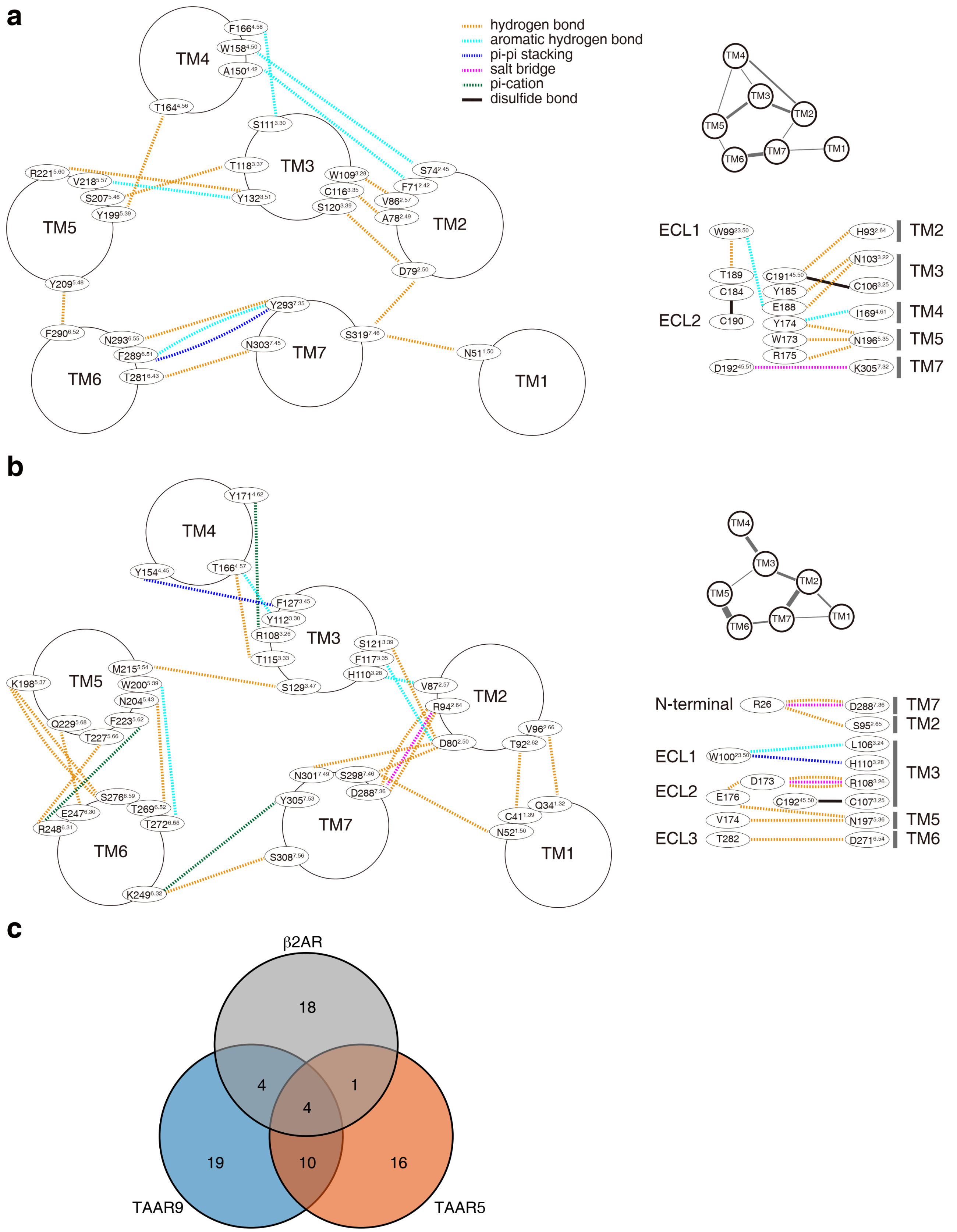

Supplementary Figure S1. TAAR9 has a higher level of conservation in intra-receptor

interaction with TAAR5 than with β2AR. (**a**) Inter-helical non-covalent interactions and interactions with ECLs in β2AR. The similar overall inter-helical interaction pattern in β2AR comparing with that in TAAR9, without interactions between TM1 and TM2. Residues interacting with extracellular domains located in TM2, 3, 4, 5, 7 of β2AR, and TM1, 2, 3, 5, 6 of TAAR9. (**b**) Inter-helical non-covalent interactions and interactions with ECLs in TAAR5. Apart from interactions that same as Fig. 1b, pi-cation interaction (green) also exists. TM4 only interact with TM3 in TAAR5, while it interacts with TM2, 3, 5 in TAAR9. Other interaction patterns within TMs are analogous with TAAR9. Residues in TM7 interact with extracellular domains in TAAR5 instead of residues in TM1 in TAAR9. (**c**) The level of conservation in intra-receptor interactions among TAAR5, TAAR9, and β2AR. 4 common interactions are present among these three receptors. In addition, other 10 interactions common in TAAR5 and TAAR9, 4 common in TAAR9 and β2AR, 1 common in TAAR5 and β2AR are observed. TAAR9, TAAR5, and β2AR have 18, 19, and 16 specific interactions, respectively.

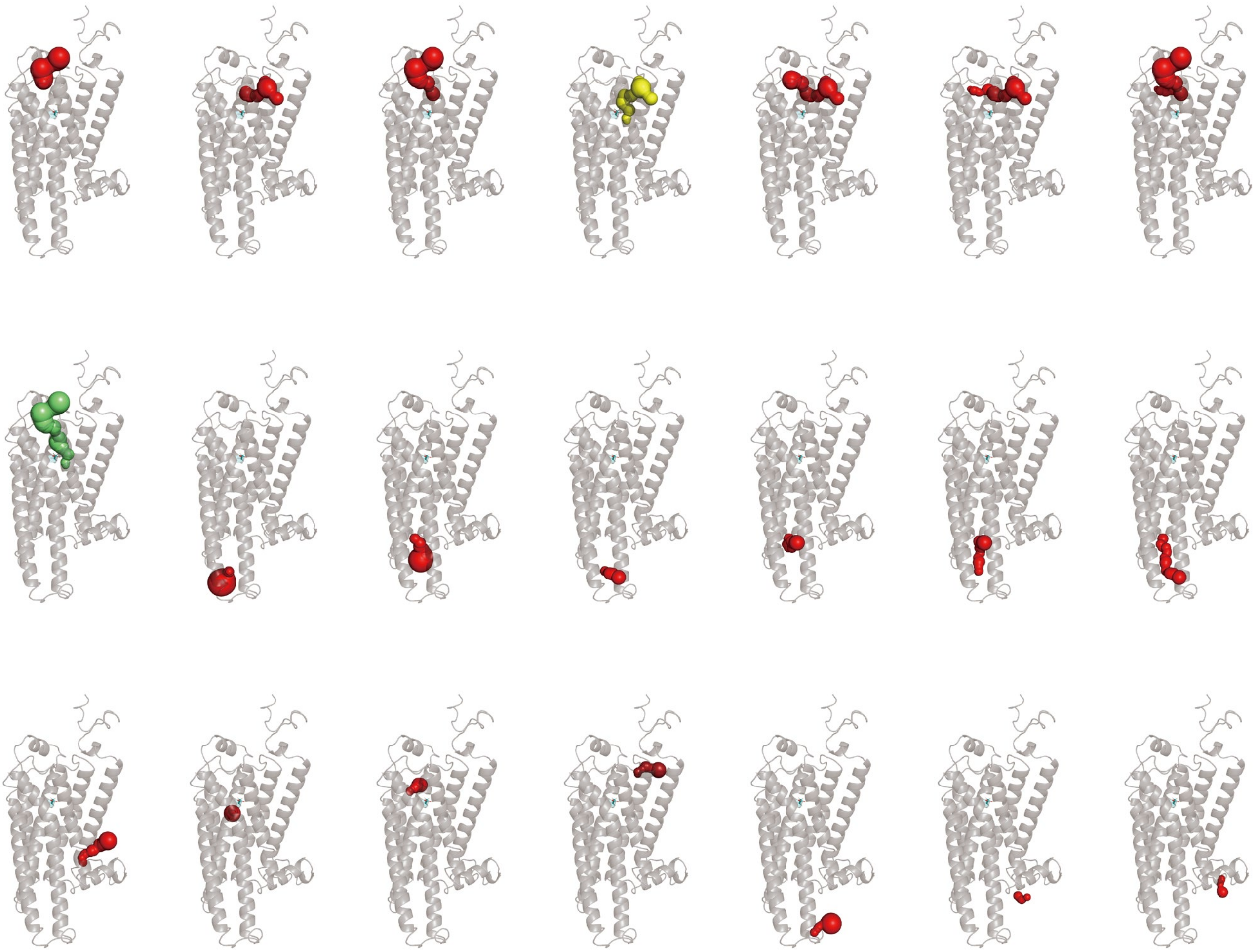

Supplementary Figure S2. 21 tunnels of TAAR9 predicted by MOLE2.5. We applied MOLE2.5 to explore intra-receptor space in TAAR9 and retrieved a result of 21 tunnels (red, green, yellow). We examined these tunnels in detail and found two tunnels which are open to the extracellular space and reach the orthosteric binding sites (blue). We named them Tunnel 1 (green) and Tunnel 2 (yellow), respectively.

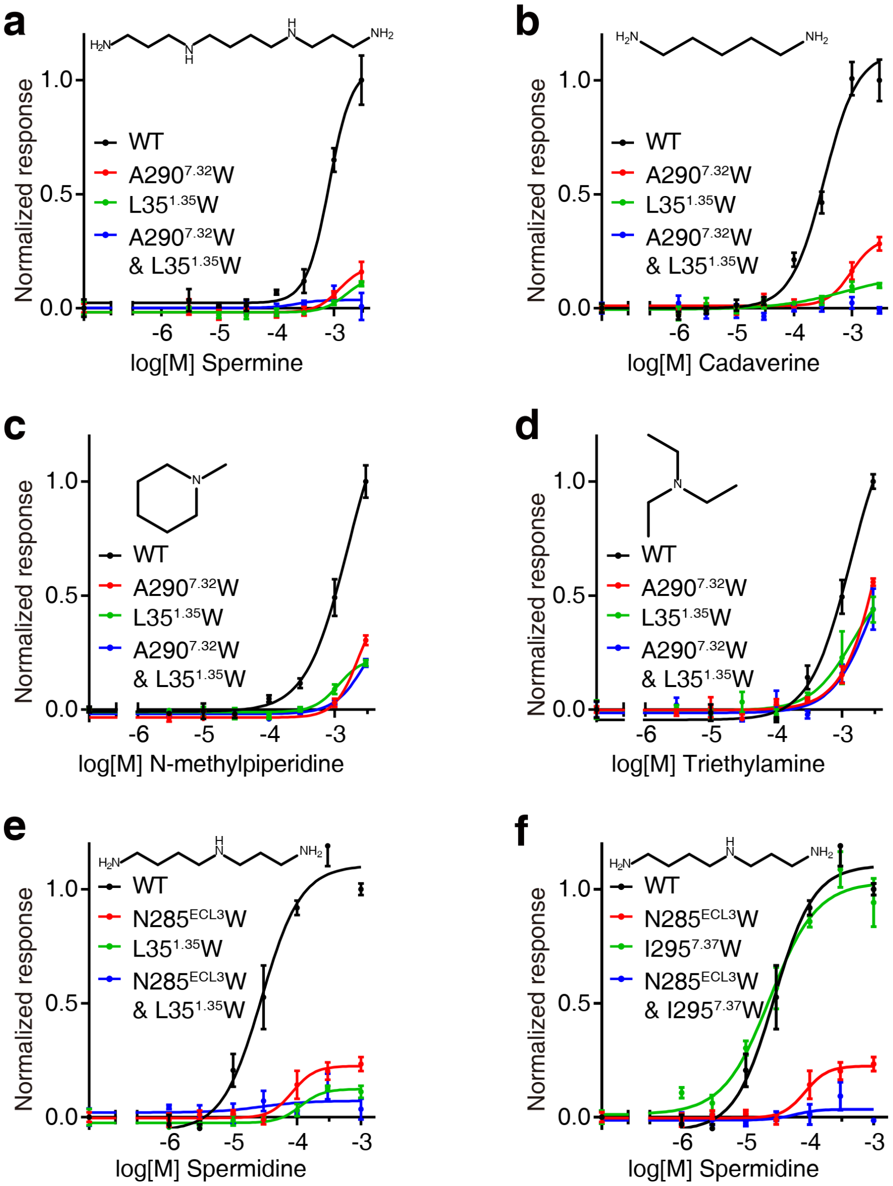

g

TAAR9	Maximal response	EC_{50} (M)
WT	1.105	2.796×10^{-5}
N285^{ECL3}W (Tunnel 1)	0.2245	8.457×10^{-5}
L35$^{1.35}$W (Tunnel 2)	0.1236	1.139×10^{-4}
I295$^{7.37}$W (Tunnel 2)	1.032	2.397×10^{-5}
N285^{ECL3}W & I295$^{7.37}$W	-	-
N285^{ECL3}W & L35$^{1.35}$W	-	-

Supplementary Figure S3. Responses of additional TAAR9 mutants to other TAAR9 ligands. (**a-d**) Dose-dependent response curves of WT, A290$^{7.32}$W single mutant, L35$^{1.35}$W single mutant, and A290$^{7.32}$W & L35$^{1.35}$W double mutant TAAR9 to four ligands, including spermine (**a**), cadaverine (**b**), N-methylpiperidine (**c**), and triethylamine (**d**). (**e**) Dose-dependent response curves of WT, N285^{ECL3}W

single mutant, L35$^{1.35}$W single mutant, and N285^{ECL3}W & L35$^{1.35}$W double mutant TAAR9 to spermidine. (**f**) Dose-dependent response curves of WT, N285^{ECL3}W single mutant, I295$^{7.37}$W single mutant, and N285^{ECL3}W & I295$^{7.37}$W double mutant TAAR9 to spermidine. (**h**) Summary of maximal responses and EC_{50} values of the single mutant of Tunnel 1 (N285^{ECL3}W), two single mutants of Tunnel 2 (L35$^{1.35}$W and I295$^{7.37}$W), and two double mutants (N285^{ECL3}W & I295$^{7.37}$W and N285^{ECL3}W & L35$^{1.35}$W).

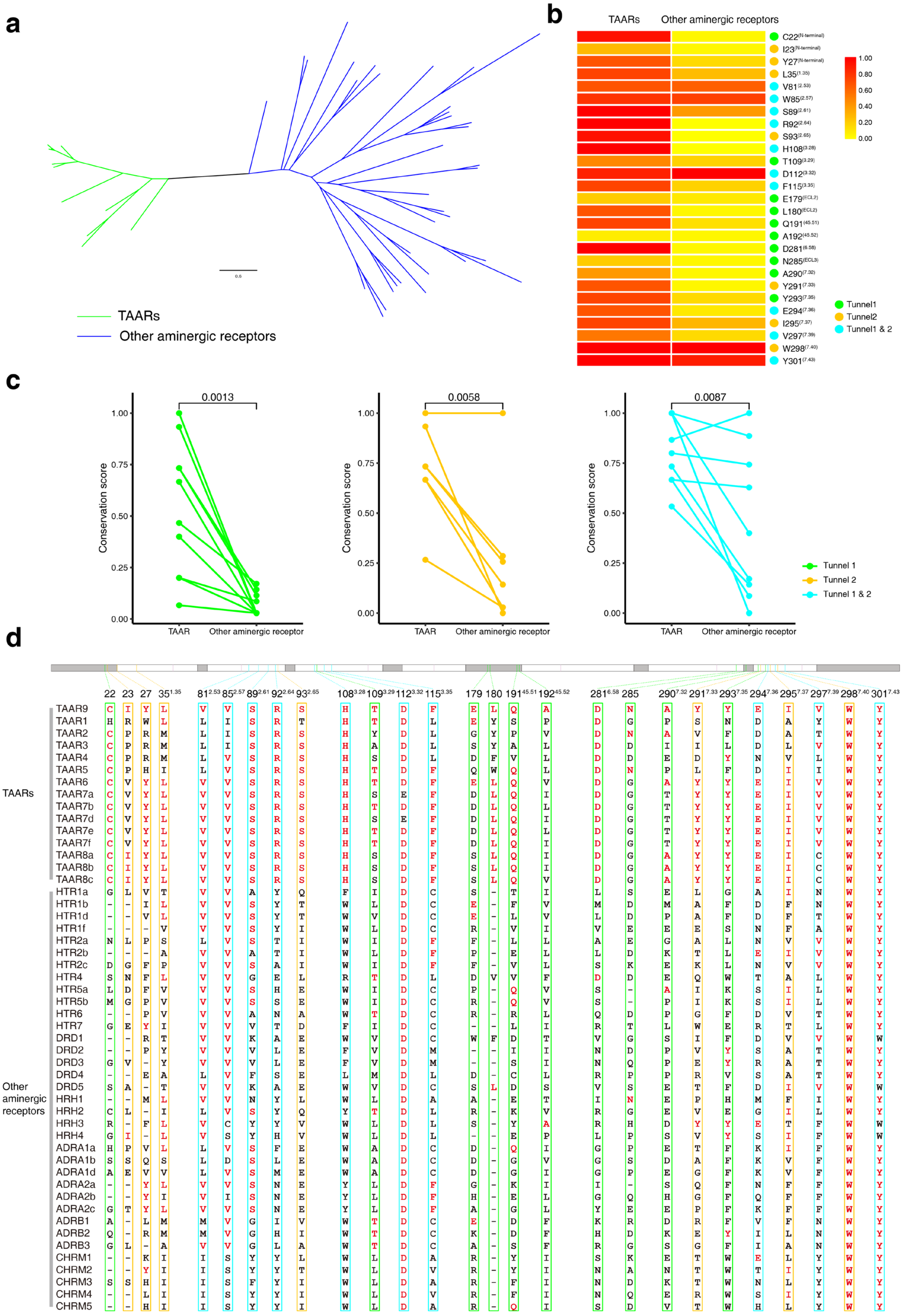
a
TAARs
Other aminergic receptors
0.5
b
TAARs
Other aminergic receptors
C22(N-terminal)
I23(N-terminal)
Y27(N-terminal)
L35(1.35)
V81(2.53)
W85(2.57)
S89(2.61)
R92(2.64)
S93(2.65)
H108(3.28)
T109(3.29)
D112(3.32)
F115(3.35)
E179(ECL2)
L180(ECL2)
Q191(45.51)
A192(45.52)
D281(6.58)
N285(ECL3)
A290(7.32)
Y291(7.33)
Y293(7.35)
E294(7.36)
I295(7.37)
V297(7.39)
W298(7.40)
Y301(7.43)
1.00
0.80
0.60
0.40
0.20
0.00
Tunnel1
Tunnel2
Tunnel1 & 2
c
Conservation score
0.0013
0.0058
0.0087
TAAR
Other aminergic receptor
Tunnel 1
Tunnel 2
Tunnel 1 & 2
d
TAARs
Other aminergic receptors
TAAR9
TAAR1
TAAR2
TAAR3
TAAR4
TAAR5
TAAR6
TAAR7a
TAAR7b
TAAR7d
TAAR7e
TAAR7f
TAAR8a
TAAR8b
TAAR8c
HTR1a
HTR1b
HTR1d
HTR1f
HTR2a
HTR2b
HTR2c
HTR4
HTR5a
HTR5b
HTR6
HTR7
DRD1
DRD2
DRD3
DRD4
DRD5
HRH1
HRH2
HRH3
HRH4
ADRA1a
ADRA1b
ADRA1d
ADRA2a
ADRA2b
ADRA2c
ADRB1
ADRB2
ADRB3
CHRM1
CHRM2
CHRM3
CHRM4
CHRM5

Supplementary Figure S4. Conservation of residues along two tunnels of TAAR9 in aminergic receptors. (**a**) Radical layout of phylogenetic tree of 50 aminergic receptors. TAAR family is clearly delineated from other aminergic receptors. (**b**) Conservation of 27 residues along two tunnels of TAAR9 among TAAR family and other aminergic receptors. The level of conservation of a specific site was calculated by the ratio of receptor having this site to the total number of receptors. (**c**) Pairwise comparison of conservation between TAAR and other aminergic receptors. Residues specific to Tunnel 1 and Tunnel 2 or common to two tunnels all show significant differences ($p < 0.05$) between TAAR and other aminergic receptors. (**d**) Details of all 27 residues in each receptor. These residues are more conserved in TAARs than in other receptors, except for $D^{3.32}$, $W^{7.40}$, and $Y^{7.43}$. Some residues are specific in TAARs, such as $R^{2.64}$, $S^{2.65}$, and $H^{3.28}$.

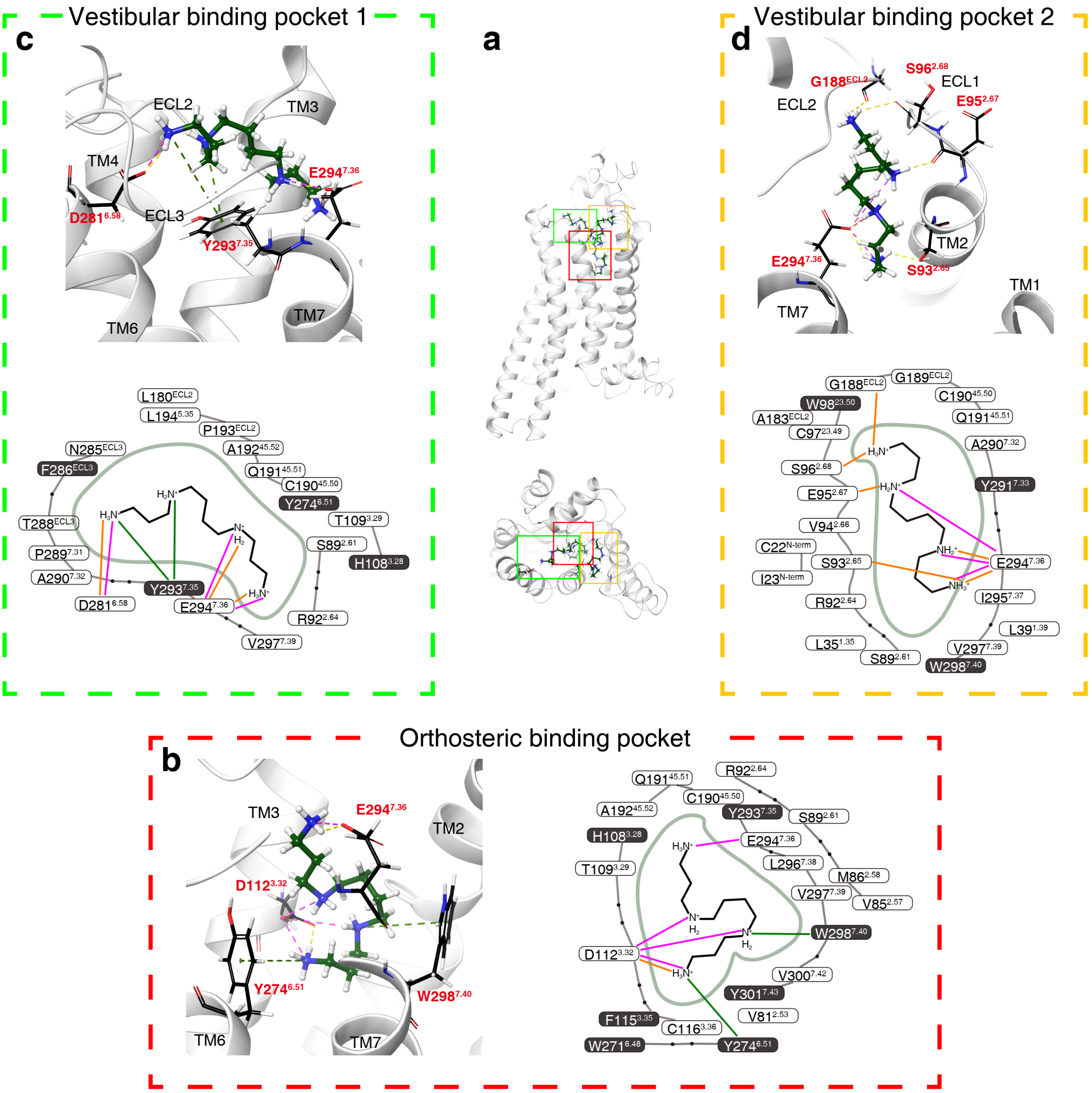

Supplementary Figure S5. Binding poses of spermine predicted by multistep induced-fit docking. (**a**) Docking of another TAAR9 ligand, spermine, into the receptor revealed three similar binding pockets as those described in Fig. 4a. (**b**) Spermine forms non-covalent bonds with residues in the orthosteric binding pocket in a similar pattern to spermidine. All binding sites of spermidine are involved in binding of spermine. (**c**) Spermine can also be docked into vestibular binding pocket 1 and forms salt bridge with D281$^{6.58}$. 16 residues surrounding spermidine in vestibular binding pocket 1 are also observed in the docking posture of spermine. (**d**) Spermine can also be docked into vestibular binding pocket 2, interacting with E294$^{7.36}$, S93$^{2.65}$, S96$^{2.68}$, and G188 in ECL2. Those residues are

also observed to function as binding sites of spermidine in vestibular binding pocket 2. The only difference is that the extracellular residue, E95$^{2.67}$, instead of Q191$^{45.51}$ in ECL2 is observed in spermine binding. 23 residues of vestibular binding pocket 2 are within 5 Å range of spermine, that include all 19 residues within 5 Å range of spermidine.

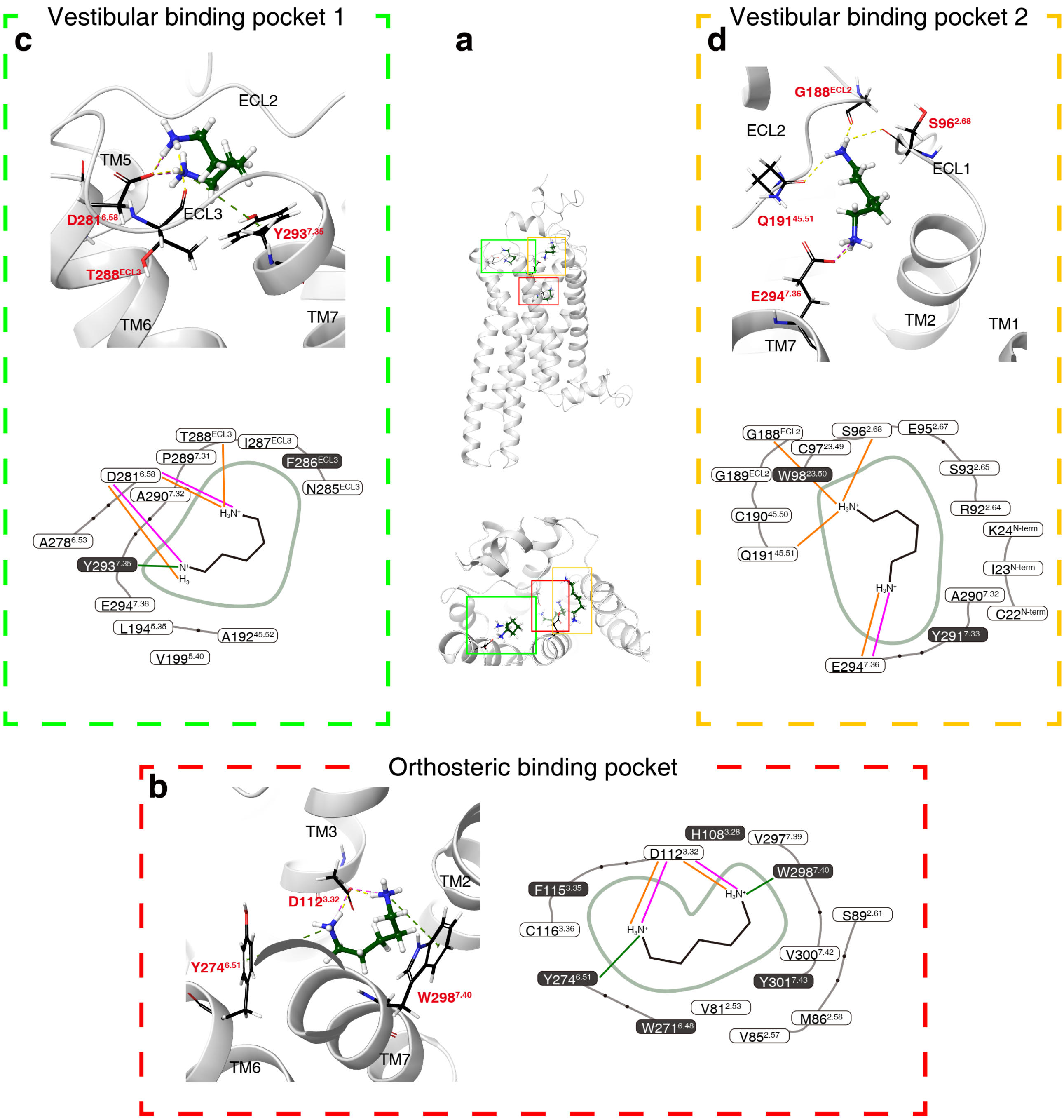

Supplementary Figure S6. Binding poses of cadaverine predicted by multistep induced-fit docking. (**a**) Cadaverine, another TAAR9 ligand, was also docked into three similar binding pockets as those described in Fig. 4a. (**b**) Cadaverine binding pose in the orthosteric binding pocket is similar to spermidine and spermine. Cadaverine can interact with $D112^{3.32}$, but not with $E294^{7.36}$. Except for $Y293^{7.35}$, other residues with aromatic rings which are within 5 Å range of spermidine also exist in docking result of cadaverine. 13 of 14 residues within 5 Å range of cadaverine are the same as those

in the docking of spermidine. (**c**) Cadaverine can form salt bridges and hydrogen bonds with D281$^{6.58}$ in vestibular binding pocket 1. Other interactive residues in TAAR9 including Y293$^{7.35}$ and T288 in ECL3 are also found in docking of spermidine. 11 of 13 residues located within 5 Å range of cadaverine are the same as spermidine in vestibular binding pocket 1. (**d**) Cadaverine can also bind to E294$^{7.36}$ in vestibular binding pocket 2. Hydrogen bonds with three residues, S96$^{2.68}$, G188 in ECL2, and Q191$^{45.51}$ in ECL2, that are observed in docking results of cadaverine also exist in the docking results of spermidine. 14 of 16 residues located within 5 Å range of cadaverine are observed in docking of spermidine in vestibular binding pocket 2.

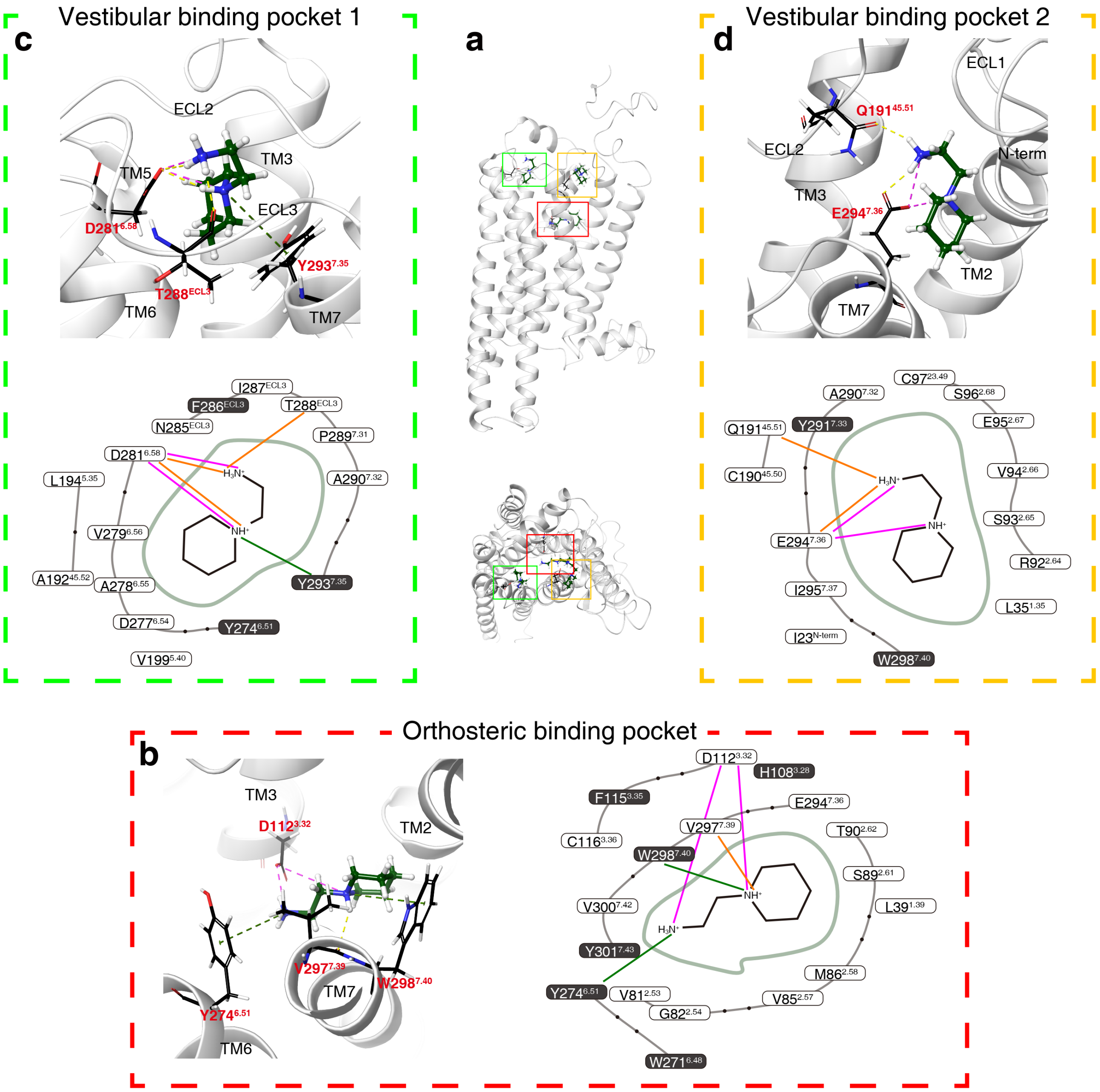

Supplementary Figure S7. Binding poses of 1-(2-aminoethyl)piperidine predicted by multistep induced-fit docking. (**a**) Docking of 1-(2-aminoethyl)piperidine into TAAR9 shows three similar binding pockets as those described in Fig. 4a. (**b**) 1-(2-aminoethyl)piperidine can be docked to orthosteric binding pocket which is in common with cadaverine. Another residue, V297$^{7.39}$, was also noted to function in binding 1-(2-aminoethyl)piperidine. 14 of 18 residues located within 5 Å range of 1-(2-aminoethyl)piperidine are the same as those in the docking of spermidine. (**c**) 1-(2-aminoethyl)piperidine can interact with D281$^{6.58}$, Y293$^{7.35}$, and T288 in ECL3 in vestibular binding pocket 1. Another aromatic ring, Y274$^{6.51}$, which is not observed in docking result of spermidine, exist

in that of 1-(2-aminoethyl)piperidine. 15 residues are located within 5 Å range of 1-(2-aminoethyl)piperidine in vestibular binding pocket 1. (**d**) 1-(2-aminoethyl)piperidine can bind to E294$^{7.36}$ in vestibular binding pocket 2. It can also form hydrogen bond with Q191$^{45.51}$ in ECL2. All of the 15 residues located within 5 Å range of 1-(2-aminoethyl)piperidine in vestibular binding pocket 2 are the same as those in the docking of spermidine.

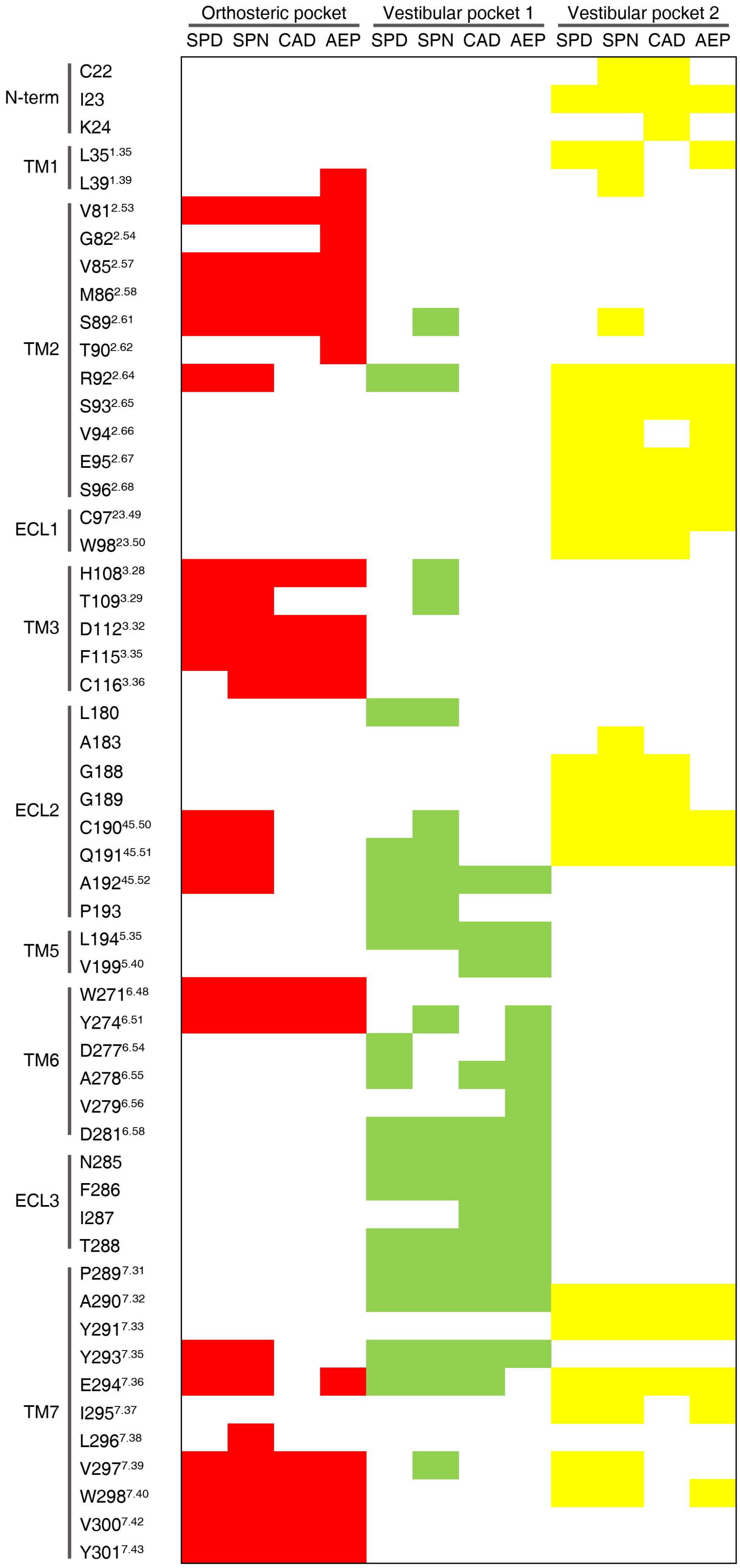
Orthosteric pocket
Vestibular pocket 1
Vestibular pocket 2
SPD SPN CAD AEP SPD SPN CAD AEP SPD SPN CAD AEP
N-term
C22
I23
K24
TM1
L35 1.35
L39 1.39
TM2
V81 2.53
G82 2.54
V85 2.57
M86 2.58
S89 2.61
T90 2.62
R92 2.64
S93 2.65
V94 2.66
E95 2.67
S96 2.68
ECL1
C97 23.49
W98 23.50
TM3
H108 3.28
T109 3.29
D112 3.32
F115 3.35
C116 3.36
ECL2
L180
A183
G188
G189
C190 45.50
Q191 45.51
A192 45.52
P193
TM5
L194 5.35
V199 5.40
TM6
W271 6.48
Y274 6.51
D277 6.54
A278 6.55
V279 6.56
D281 6.58
ECL3
N285
F286
I287
T288
TM7
P289 7.31
A290 7.32
Y291 7.33
Y293 7.35
E294 7.36
I295 7.37
L296 7.38
V297 7.39
W298 7.40
V300 7.42
Y301 7.43

Supplementary Figure S8. Most of the residues within 5 Å of the orthosteric binding pocket of four ligands are in common. Presence of the critical residues within 5 Å of the orthosteric binding pocket (red), vestibular pocket 1 (green), and vestibular pocket 2 (yellow) are shown from the docking results of four TAAR9 ligands. 13 residues in the orthosteric binding pocket are among docking results of four ligands are in common. C116$^{3.36}$ and E294$^{7.36}$ are common among docking results of three ligands. These 15 residues are defined as the residues constituting the orthosteric binding pocket. 6 residues, including R92$^{2.64}$ and 3 residues in ECL2, are common in spermidine and sperminine docking results. Other 4 residues are specific for one ligand docking. In vestibular binding pocket 1, the numbers of residues common for docking results of 4 ligands, 3 ligands, 2 ligands, and 1 ligand are 9, 2, 9, and 5. Hence, there are 11 residues considered as the residues constituting vestibular binding pocket 1. In vestibular binding pocket 2, the numbers of residues common for docking results of 4 ligands, 3 ligands, 2 ligands, and 1 ligand are 11, 7, 2, and 4. Hence, there are 18 residues considered as the residues constituting vestibular binding pocket 2. SPD, spermidine; SPN, spermine; CAD, cadaverine; AEP, 1-(2-aminoethyl)piperidine.

a

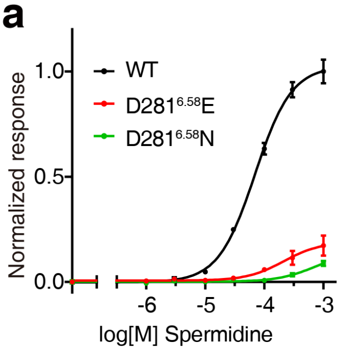

TAAR9	Maximal response	EC_{50} (M)
WT	1.027	6.972×10^{-5}
D281$^{6.58}$E	0.1896	2.12×10^{-4}
D281$^{6.58}$N	0.1411	7.05×10^{-4}

b

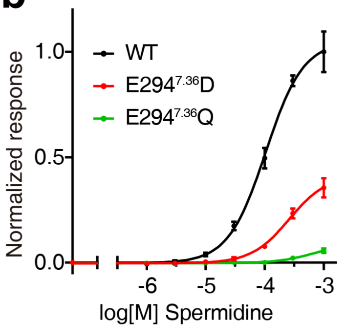

TAAR9	Maximal response	EC_{50} (M)
WT	1.051	1.041×10^{-4}
E294$^{7.36}$D	0.4134	2.581×10^{-4}
E294$^{7.36}$Q	0.0936	7.089×10^{-4}

Supplementary Figure S9. Mutations of residues in vestibular binding pockets lead to changes of ligand recognition. (**a**) Maximal responses and EC_{50} values of mutations in D281$^{6.58}$ in Fig. 4e. The D281$^{6.58}$E mutant shows lower response compared with WT TAAR9, but larger response compared with the D281$^{6.58}$N. (**b**) Maximal response and EC_{50} values of mutations in E294$^{7.36}$ in Fig. 4g. The E294$^{7.36}$D mutant shows less than 3-fold decreased response compared with WT. However, little response can be observed for E294$^{7.36}$Q mutant.

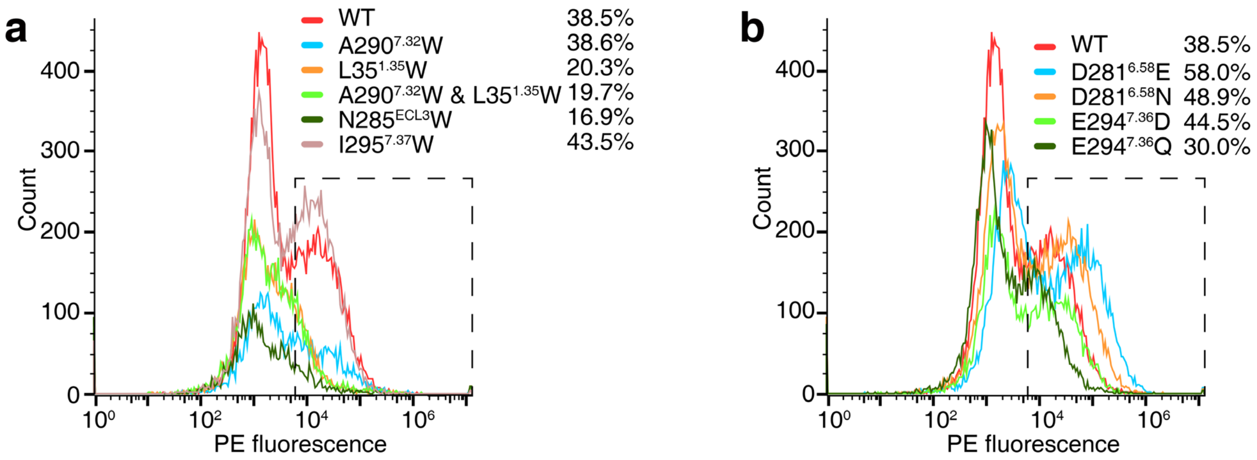

Supplementary Figure S10. Analysis of cell-surface expression by FACS. Hana3A cells expressing WT or mutant TAAR9 with EGFP. WT and mutant TAAR9 receptors have Rho tag in their N-terminus, which can be used for surface labeling. The receptors expressed on the cell surface are marked by dotted box, and the percentages of receptors on the cell surface are labeled on the corner. (**a**) The A290$^{7.32}$W and I295$^{7.37}$W mutants have similar cell-surface expression levels compared to WT. The L35$^{1.35}$W, A290$^{7.32}$W & L35$^{1.35}$W, and N285^{ECL3}W mutants show slightly decreased cell-surface expression levels to WT. (**b**) The D281$^{6.58}$E, D281$^{6.58}$N, and E294$^{7.36}$D mutants show increased cell-surface expression levels to WT, while the E294$^{7.36}$Q mutant shows slightly decreased cell-surface expression levels to WT.

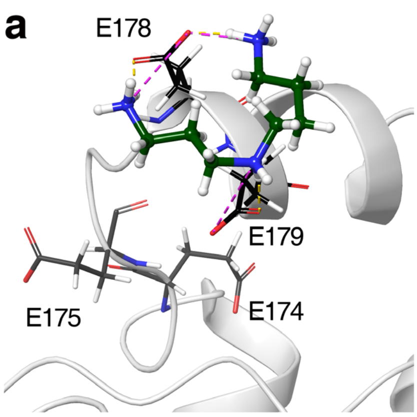

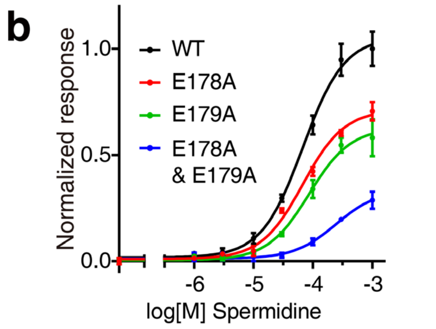

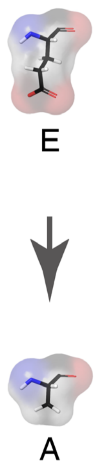

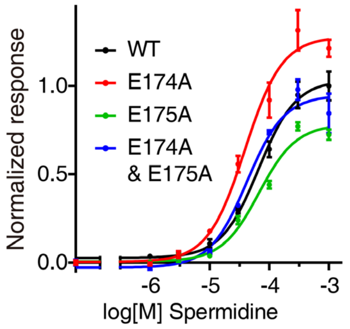

TAAR9	Maximal response	EC_{50} (M)
WT	1.056	6.792×10^{-5}
E178A	0.713	6.746×10^{-5}
E179A	0.6281	8.619×10^{-5}
E178A & 179A	0.3358	2.444×10^{-4}

TAAR9	Maximal response	EC_{50} (M)
WT	1.032	6.62×10^{-5}
E174A	1.277	3.922×10^{-5}
E175A	0.7819	6.709×10^{-5}
E174A & 175A	0.9472	4.241×10^{-5}

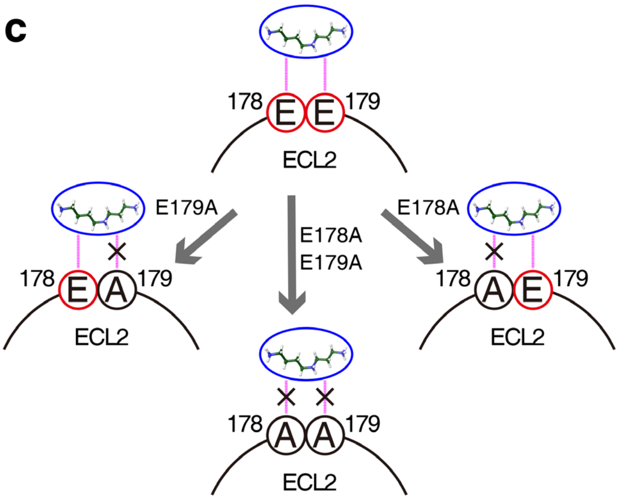

Supplementary Figure S11. Elimination of negative charge of some glutamate residues in ECL2 of TAAR9 decreases its ligand binding efficacy. (**a**) Two pairs of adjacent glutamates are present in ECL2. E178 and E179 in ECL2 are observed to interact with spermidine in the docking

model. (**b**) E178 and E179 were mutated to A (left) separately or simultaneously. Single mutants show slightly decreased responses but similar EC_{50}. However, double mutant gives rise to a much lower maximal response and larger EC_{50}. E174 and E175 were mutated to A (right) separately or simultaneously. All of the three mutants show comparable maximal responses and EC_{50} with wild type TAAR9. (**c**) Schematic illustration of ligand binding to ECL2 of TAAR9. Both of E178 and E179 can bind to spermidine through salt bridge and recruit the ligand to the tunnels. Elimination of any glutamate can be compensated by the other one.

Tunnel number	Lining residues
predicted tunnel 1	C22, E179, L180, Q191, A192, L194, D281, N285, A290, Y293
predicted tunnel 2	I23, Y27, L35, S89, R92, S93, H108, C190, A192, Y291, E294, I295, V297, W298
predicted tunnel 3	C22, R92, H108, E179, L180, C190, Q191, A192, D281, N285, A290, Y293, E294, V297
predicted tunnel 4 (Tunnel 2)	I23, Y27, L35, V81, V85, S89, R92, S93, H108, D112, F115, Y291, E294, I295, V297, W298, Y301
predicted tunnel 5	I23, Y27, L35, S89, R92, S93, H108, Q191, A192, L194, D281, A290, Y291, Y293, E294, I295, V297, W298
predicted tunnel 6	I23, Y27, L35, S89, R92, S93, H108, T109, F168, N173, C190, Q191, A192, P193, W198, Y291, E294, I295, V297, W298
predicted tunnel 7	C22, R92, H108, T109, F168, N173, E179, L180, C190, Q191, A192, P193, W198, D281, N285, A290, Y293, E294, V297
predicted tunnel 8 (Tunnel 1)	C22, V81, V85, S89, R92, H108, T109, D112, F115, E179, L180, Q191, A192, D281, N285, A290, Y293, E294, V297, Y301
predicted tunnel 9	L138, Q227, K230, I231, K246, V249, A250
predicted tunnel 10	R130, A133, V134, P137, Y140, I220, V249, E253, A256, A257
predicted tunnel 11	Q227, A228, I231, E232, S241, E247, A250, R254
predicted tunnel 12	R130, V134, I220, F221, A224, E253, R254, A257, K258
predicted tunnel 13	F221, A224, Q227, A228, I231, A250, E253, R254, A257, K258,
predicted tunnel 14	R130, V134, I220, A224, Q227, A228, I231, E232, S241, E247, A250, E253, R254, A257
predicted tunnel 15	L45, F48, G49, L52, V53, P308, L309, Y311, A312, F318, I322
predicted tunnel 16	F111, S114, W157, V161
predicted tunnel 17	S103, K106, F107, C110, F165, F168, Y169,
predicted tunnel 18	V91, R92, V94, E95, W98, Y99, F100,
predicted tunnel 19	S241, S242, E243, S244, E247, R248, K251,
predicted tunnel 20	Q62, W317, K320
predicted tunnel 21	K320, A321, L324, T338, A345, G346

Table S1. Residues in the 21 tunnels predicted by MOLE2.5. The lining residues around 21 predicted tunnels are listed in the same order as the Supplementary Fig. S2 online. Only predicted tunnel 4 (named Tunnel2) and predicted tunnel 8 (named Tunnel1) pass through $D112^{3.32}$.

Location	Tunnel 1	Tunnel 2
N-term	C22	
		I23
		Y27
TM1		$L35^{1.35}$
TM2	$V81^{2.53}$	$V81^{2.53}$
	$V85^{2.57}$	$V85^{2.57}$
	$S89^{2.61}$	$S89^{2.61}$
	$R92^{2.64}$	$R92^{2.64}$
		$S93^{2.65}$
TM3	$H108^{3.28}$	$H108^{3.28}$
	$T109^{3.29}$	
	$D112^{3.32}$	$D112^{3.32}$
	$F115^{3.35}$	$F115^{3.35}$
ECL2	E179	
	L180	
	$Q191^{45.51}$	
	$A192^{45.52}$	
TM6	$D281^{6.58}$	
ECL3	N285	
TM7	$A290^{7.32}$	
		$Y291^{7.33}$
	$Y293^{7.35}$	
	$E294^{7.36}$	$E294^{7.36}$
		$I295^{7.37}$
	$V297^{7.39}$	$V297^{7.39}$
		$W298^{7.40}$
	$Y301^{7.43}$	$Y301^{7.43}$

Table S2. Residues around two tunnels. The specific location of residues around Tunnel 1 and Tunnel 2 are listed in order. Residues in TM2, TM3, TM7 account for most composition.

Property	Value	
	Tunnel 1	Tunnel 2
Length (Å)	35.9	32.9
Bottleneck (Å)	1.3	1.3
Hydropathy	-0.4	-0.13
Charge	-3	-1
Polarity	14.76	16.45
Mutability	82	79
Ionizable	5	3

Table S3. Values of properties of two tunnels offered by MOLE website. Both tunnels show similar properties in length, bottleneck, polarity, mutability. Tunnel 1 is more hydrophilic than Tunnel 2. The number of ionizable residues of Tunnel 1 and Tunnel 2 is 5 and 3, respectively, which leads to a more negative charge of Tunnel 1. Length, length of the tunnel. Bottleneck, radius of the narrowest neck of the tunnel. Hydropathy, average of hydropathy index per each amino acid. Polarity, average of lining amino acid polarities using statistical methods by Zimmermann et al[1]. Mutability, average of relative mutability index based on empirical substitution matrices between similar protein sequences[2]. Ionizable, number of ionizable residues

1 Zimmerman, J. M., Eliezer, N. & Simha, R. The characterization of amino acid sequences in proteins by statistical methods. *J Theor Biol* **21**, 170-201, doi:10.1016/0022-5193(68)90069-6 (1968).

2 Jones, D. T., Taylor, W. R. & Thornton, J. M. The rapid generation of mutation data matrices from protein sequences. *Comput Appl Biosci* **8**, 275-282, doi:10.1093/bioinformatics/8.3.275 (1992).

Location	Tunnel 1	Tunnel 2	Properties of the residues
N-term	C22		In dynamic N terminal
		I23	In dynamic N terminal
		Y27	In dynamic N terminal
TM1		$L35^{1.35}$	-
TM2		$S93^{2.65}$	Interacting with $R32^{1.32}$ forming hydrogen bond
TM3	$T109^{3.29}$		Around orthosteric binding pocket
ECL2	E179		In dynamic ECL2
	L180		In dynamic ECL2
	$Q191^{45.51}$		In ECL2 and around orthosteric binding pocket
	$A192^{45.52}$		In ECL2 and around orthosteric binding pocket
TM6	$D281^{6.58}$		Negative charged
ECL3	N285		In ECL3 but near TM6
TM7	$A290^{7.32}$		-
		$Y291^{7.33}$	Steric hindrance is already high
	$Y293^{7.35}$		Steric hindrance is already high, and around orthosteric binding pocket
		$I295^{7.37}$	-
		$W298^{7.40}$	Steric hindrance is already high, and around orthosteric binding pocket

Table S4. Locations and features of 17 residues specific to each tunnel. Eight of the specific residues are located in dynamic extracellular regions. Five of them are near orthosteric binding pocket. $S93^{2.65}$ is involved in the intra-receptor interaction. $D281^{6.58}$ is negatively-charged. $Y291^{7.33}$, $Y293^{7.35}$, and $W298^{7.40}$ have high steric hindrance. Therefore, the above-mentioned residues were not selected for mutagenesis study. We selected $L35^{1.35}$, $A290^{7.32}$, and $I295^{7.37}$ to generate TAAR9 mutants. Besides, N285 in ECL3 was also selected as it is near TM6 and its position is relatively fixed.

Supplementary Data. Amino acid sequences of 50 mouse aminergic receptors. 50 aminergic receptor protein sequences retrieved form NCBI are presented in Fasta format. Accession numbers are added after abbreviation of the receptors in the unique sequence identifier.

>MmTaar1-NP_444435.1
MHLCHAITNISHRNSDWSREVQASLYSLMSLIILATLVGNLIVIISISHFKQLHTPTNWLLHSMAI
VDFLLGCLIMPCSMVRTVERCWYFGEILCKVHTSTDIMLSSASIFHLAFISIDRYCAVCDPLRY
KAKINISTILVMILVSWSLPAVYAFGMIFLELNLKGVEELYRSQVSDLGGCSPFFSKVSGVLAF
MTSFYIPGSVMLFVYYRIYFIAKGQARSINRTNVQVGLEGKSQAPQSKETKAAKTLGIMVGVF
LVCWCPFFLCTVLDPFLGYVIPPSLNDALYWFGYLNSALNPMVYAFFYPWFRRALKMVLLGK
IFQKDSSRSKLFL

>MmTaar2-NP_001007267.1
MASFEAQQETFDCSEYGNGSCPENERSLGVRAAMYSLMACAIFITIFGNLAMIISISYFKQLHT
PTNLLILSMAVTDFLLGFTIMPYSMVRSVENCWYFGLTFCKIHYSFDLMLSITSIFHLCSVAVD
RFYAICHPLHYCTKMTIPVVRRLLLVCWSVPGAFAFGVVFSEAYADGIEGYDILVACSSSCPV
MFNKLWGTTLFVAGFFTPSSMMVGIYGKIFAVSKKHARVIDNLPENQNNQMRKDKKAAKTL
GIVMGVFLLCWFPCFFTILLDPFLNFSTPAVLFDALTWFGYFNSTCNPLIYGFFYPWFRRALK
YILLGKIFSSHFHNTNLFTQKETE

>MmTaar3-NP_001008429.1
MDLIYIPEDLSSCPKFGNKSCPPTNRSFRVRMIMYLFMTGAMVITIFGNLVIIISISHFKQLHSPT
NFLILSMATTDFLLGFVIMPYSMVRSVESCWYFGDSFCKFHASFDMMLSLTSIFHLCSIAIDRF
YAVCDPLHYTTTMTVSMIKRLLAFCWAAPALFSFGLVLSEANVSGMQSYEILVACFNFCALTF
NKFWGTILFTTCFFTPGSIMVGIYGKIFIVSRRHARALSDMPANTKGAVGKNLSKKKDRKAAK
TLGIVMGVFLACWLPCFLAVLIDPYLDYSTPIIVLDLLVWLGYFNSTCNPLIHGFFYPWFRKAL
QFIVSGKIFRSNSDTANLFPEAH

>MmTaar4-NP_001008499.1
MNTPDPWSSPEVQFCFAAANSSCPRKARPALVVCAMYLIMIGAIVMTMLGNMAVIISIAHFKQ
LHSPTNFLILSMATTDFLLSCVVMPFSMIRSIESCWYFGDLFCKVHSCCDIMLCTTSIFHLCFIS
VDRHYAVCDPLHYVTQITTRVVGVFLLISWSVPIFFAFGLVFSELNLIGAEDFVAAIDCTGLCVL
IFNKLWGVLASFIAFFLPGTVMVGIYIHIFTVAQKHARQIGTGPRTKQALSESKMKATSKKESK
ATKTLSIVMGVFVLCWLPFFVLTITDPFIDFTTPEDLYNVFLWLGYFNSTFNPIIYGMFYPWFR
KALRMIVTGTIFRSDSSTSSLHPAHP

>MmTaar5-NP_001009574.1
MRAVLLPGSGEQPTAFCYQVNGSCPRTVHPLAIQVVIYLACAVGVLITVLGNLFVVFAVSYFK
VLHTPTNFLLLSLALADMLLGLLVLPLSTVRSVESCWFFGDFLCRLHTYLDTLFCLTSIFHLCFI
SIDRHCAICDPLLYPSKFTVRTALRYIVAGWGIPAAYTAFFLYTDVVERALSQWLEEMPCVGS
CQLLFNKFWGWLNFPAFFVPCLIMISLYLKIFVVATRQAQQIRTLSQSLAGAVKRERKAAKTL
GIAVGIYLVCWLPFTVDTLVDSLLNFITPPLVFDIFIWFAYFNSACNPIIYVFSYRWFRKALKLLL
SREIFSPRTPTVDLYHD

>MmTaar6-NP_001010828.1
MGSNSSPPTVLQLCYENVTGSCVKTPYSPGSRVILYAVFGFGAVLAVFGNLMVMISILHFKQL
HSPTNFLIASLACADFGVGISVMPFSMVRSIESCWYFGRSFCTFHTCCDVAFCYSSLFHLSFI
SIDRYIAVTDPLVYPTKFTVSVSGICIGVSWILPLVYSGAVFYTGVYDDGLEELSSALNCVGGC
QVVVNQNWVLIDFLSFLIPTLVMIILYGNIFLVARQQAKKIENIGSKTESSSESYKARVARRERK
AAKTLGITVVAFMISWLPYSIDSLVDAFMGFITPAYIYEICVWCAYYNSAMNPLIYALFYPWFK
KAIKVIMSGQVFKNSSATMNLFSEQI

>MmTaar7a-NP_001010829.1
MDKLVDHFLSDQSRTMNEDLFSATSTELCYENLNRSCVRSPYSPGPRLILYAVFGFGAALAV
CGNLLVMTSILHFRQLHSPANFLVASLACADFLVGLTVMPFSTVRSVEGCWYFGESYCKFHS
CFEGSFCYSSIFHLCFISVDRYIAVSDPLTYPTRFTASVSGKCITFSWLLSIIYSFSLLYTGANE
AGLEDLVSVLTCVGGCQIAVNQSWVFINFLLFLIPTLVMMTVYSKIFLIAKQQAQNIEKMSKQT
ARASESYKDRVAKRERKAAKTLGIAVAAFLLSWLPYFIDSIIDAFLGFITPTYVYEILVWIAYYNS
AMNPLIYAFFYPWFRKAIKLIVTGKILRENSSTTNLFPE

>MmTaar7b-NP_001010827.1
MATDNDSFPWDQDSILSSDMFSATSTELCYENLNRSCVRSPYSPGPRLILYAVFGFGAALAV
CGNLLVMTSILHFRQLHSPANFLVVSLACADFLVGLTVMPFSTVRSVEGCWYFGESYCKLHT
CFDVSFCYCSIFHLCFISVDRYIAVSDPLTYPTRFTAFVSGKCITFSWLLSTIYGFSLLYTGANE
AGLEDLVSALTCVGGCQLAVNQSWVFINFLLFLIPTLVMITVYSKIFLIAKQQAQNIEKMSKQT
ARASDSYKDRVAKRERKAAKTLGIAVAAFLLSWLPYFIDSIIDAFLGFITPTYVYEILVWIAYYN
SAMNPLIYAFFYPWFRKAIKLIVSGKVLRENSSTTNLFPE

>MmTaar7d-NP_001010838.1
MATGDDSFPWDQDSILSRDLFSATSTELCYENLNRSCVRSPYSPGPRLILYAVFGFGAVLAV
CGNLLVMTSILHFRQLHSPANFLVASLACADFLVGVMVMPFSMVRSVEGCWYFGESYCKFH
SCFEGSFCYSSLFHLCFISVDRYIAVSDPLTYPTRFTASVSGKCITFSWLLSIIYSFSLLYTGAN
DAGLEDLVSALTCVGGCQIAVNQTWVFINFLLFLIPTLVMITVYSKIFLIAKQQAQNIEKMSKQT
ARASESYKDRVTKRERKAAKTLGIAVAAFLLSWLPYFIDSIIDAFLGFITPTYVYEILVWIVYYNS
AMNPLIYAFFYSWFRKAIKLIVSGKILRENSSTTNLFPE

>MmTaar7e-NP_001010835.1

MATGDDSFLWDQDSILSRDLFSATSAELCYENLNRSCVRSPYSPGPRLILYAVFGFGAVLAV
CGNLLVMTSILHFRQLHSPANFLVASLACADFLVGLTVMPFSTVRSVEGCWYFGEIYCKLHT
CFDVSFCSSSIFHLCFISVDRYIAVSDPLIYPTRFTASVSNKCITFSWLLSISYGFSLIYTGASEA
GLEDLVSALTCVGGCQLAVNQSWVFINFLLFLIPTLVMITVYSKIFLIAKQQAQNIEKMSKQTA
RASDSYKDRVAKRERKAAKTLGIAVAAFLLSWLPYFIDSFIDAFLGFITPTYVYEILVWIAYYNS
AMNPLIYAFFYPWFRKAIKLTVTGKILRENSSTTNLFPE

>MmTaar7f-NP_001010839.1

MSIADETVSWNQDSILSRDLFSATSAELCYENLNRSCVRSPYSPGPRLILYAVFGFGAVLAVC
GNLLVMTSILHFRQLHSPANFLVASLACADFLVGVMVMPFSMVRSVEGCWYFGDSYCKLHT
CFDVSFCYCSLFHLCFISVDRYIAVSDPLAYPTRFTASVSGKCITFSWLLSISYGFSLIYTGASE
AGLEDLVSSLTCVGGCQIAVNQTWVFINFSVFLIPTLVMITVYSKIFLIAKQQAQNIEKMSKQTA
RASDSYKDRVAKRERKAAKTLGIAVAAFLLSWLPYFIDSFIDAFLGFITPTYVYEILVWIVYYNS
AMNPLIYAFFYPWFRKAIKLTVTGKILRENSSTTNLFSE

>MmTaar8a-NP_001010830.1

MTSNFSQPALQLCYENTNGSCIKTPYSPGPRVILYMVYGFGAVLAVCGNLLVVISVLHFKQLH
SPANFLIASLASADFLVGISVMPFSMVRSIESCWYFGDAFCSLHSCCDVAFCYSSVLHLCFIS
VDRYIAVTDPLVYPTKFTVSVSGICISISWILPLVYSSAVFYTGISAKGIESLVSALNCVGGCQIV
INQDFVLISFLLFFIPTLVMIILYSKIFLVAKQQAVKIETSVSGNRGESSSESHKARVAKRERKAA
KTLGVTVVAFMVSWLPYTIDALVDAFMGFITPAYVYEICCWGTYYNSAMNPLIYAFFFPWFKK
AIKLILSGEILKGHSSTANLFSE

>MmTaar8b-NP_001010837.1

MTSNFSQPALQLCYENTNGSCIKTPYSPGPRVILYMVFGFGAVLAVCGNLLVVISVLHFKQLH
SPANFLIASLASADFLVGISVMPFSMVRSIESCWYFGDAFCSLHSCCDVAFCYSSALHLCFIS
VDRYIAVTDPLVYPTKFTVSVSGICISISWILPLVYSSAVFYTGISAKGIESLVSALNCVGGCQIV
VNQDWVLIDFLLFFIPTLVMIILYSKIFLVAKQQAVKIETSVSDNRGESSSESHKARVAKRERKA
AKTLGVTVVAFMVSWLPYTIDSLVDAFVGFITPAYVYEICCWSAYYNSAMNPLIYAFFYPWFR
KAIKLILSGEILKSHSSTMSLFSE

>MmTaar8c-NP_001010840.1

MTSNFSQPALQLCYENTNGSCIKTPYSPGPRVILYMVYGFGAVLAVCGNLLVVISVLHFKQLH
SPANFLIASLASADFLVGISVMPFSMVRSIESCWYFGDAFCSLHSCCDVAFCYSSALHLCFIS
VDRYIAVTDPLVYPTKFTVSVSGICISISWILPLVYSSAVFYTGISAKGIESLVSALNCVGGCQV
VVNQDWVLISFLLFFIPTVVMIILYSKIFLVAKQQAVKIETSVSGNRGESSSESHKARVAKRER
KAAKTLGVTVVAFMVSWLPYTIDALVDAFMGFITPAYVYEICCWSAYYNSAMNPLIYAFFYPW

FRKAIKLILSGKILKGHSSTTNLFSE

>MmTaar9-NP_001010831.1
MTSDFSPEPPMELCYENVNGSCIKSSYAPWPRAILYGVLGLGALLAVFGNLLVIIAILHFKQLH
TPTNFLVASLACADFLVGVTVMPFSTVRSVESCWYFGESYCKFHTCFDTSFCFASLFHLCCI
SIDRYIAVTDPLTYPTKFTVSVSGLCIALSWFFSVTYSFSIFYTGANEEGIEELVVALTCVGGC
QAPLNQNWVLLCFLLFFLPTVVMVFLYGRIFLVAKYQARKIEGTANQAQASSESYKERVAKR
ERKAAKTLGIAMAAFLVSWLPYIIDAVIDAYMNFITPAYVYEILVWCVYYNSAMNPLIYAFFYP
WFRKAIKLIVSGKVFRADSSTTNLFSEEAGAG

>MmHtr1a-NP_032334.2
MDMFSLGQGNNTTTSLEPFGTGGNDTGLSNVTFSYQVITSLLLGTLIFCAVLGNACVVAAIAL
ERSLQNVANYLIGSLAVTDLMVSVLVLPMAALYQVLNKWTLGQVTCDLFIALDVLCCTSSILHL
CAIALDRYWAITDPIDYVNKRTPRRAAALISLTWLIGFLISIPPMLGWRTPEDRSNPNECTISKD
HGYTIYSTFGAFYIPLLLMLVLYGRIFRAARFRIRKTVKKVEKKGAGTSFGTSSAPPPKKSLNG
QPGSGDCRRSAENRAVGTPCANGAVRQGEDDATLEVIEVHRVGNSKGHLPLPSESGATSY
VPACLERKNERTAEAKRKMALARERKTVKTLGIIMGTFILCWLPFFIVALVLPFCESSCHMPEL
LGAIINWLGYSNSLLNPVIYAYFNKDFQNAFKKIIKCKFCR

>MmHtr1b-NP_034612.1
MEEQGIQCAPPPPAASQTGVPLTNLSHNCSADGYIYQDSIALPWKVLLVALLALITLATTLSNA
FVIATVYRTRKLHTPANYLIASLAVTDLLVSILVMPISTMYTVTGRWTLGQVVCDFWLSSDITC
CTASIMHLCVIALDRYWAITDAVEYSAKRTPKRAAIMIVLVWVFSISISLPPFFWRQAKAEEEM
LDCFVNTDHVLYTVYSTVGAFYLPTLLLIALYGRIYVEARSRILKQTPNKTGKRLTRAQLITDSP
GSTSSVTSINSRAPDVPSESGSPVYVNQVKVRVSDALLEKKKLMAARERKATKTLGIILGAFIV
CWLPFFIISLVMPICKDACWFHMAIFDFFNWLGYLNSLINPIIYTMSNEDFKQAFHKLIRFKCAG

>MmHtr1d-NP_001272411.1
MSPPNQSLEGLPQEASNRSLNVTGAWDPEVLQALRISLVVVLSVITLATVLSNAFVLTTILLTK
KLHTPANYLIGSLATTDLLVSILVMPISIAYTTTRTWNFGQILCDIWVSSDITCCTASILHLCVIAL
DRYWAITDALEYSKRRTAGHAAAMIAAVWIISICISIPPLFWRQATAHEEMSDCLVNTSQISYTI
YSTCGAFYIPSILLIILYGRIYVAARSRILNPPSLYGKRFTTAQLITGSAGSSLCSLNPSLHESHT
HTVGSPLFFNQVKIKLADSILERKRISAARERKATKTLGIILGAFIICWLPFFVVSLVLPICRDSC
WIHPALFDFFTWLGYLNSLINPVIYTVFNEDFRQAFQKVVHFRKIS

>MmHtr1f-NP_032336.1
MDFLNASDQNLTSEELLNRMPSKILVSLTLSGLALMTTTINSLVIAAIIVTRKLHHPANYLICSLA
VTDFLVAVLVMPFSIVYIVRESWIMGQVLCDIWLSVDIICCTCSILHLSAIALDRYRAITDAVEYA
RKRTPRHAGIMITIVWVISVFISMPPLFWRHQGTSRDDECVIKHDHIVSTIYSTFGAFYIPLVLIL

ILYYKIYRAARTLYHKRQASRMIKEELNGQVFLESGEKSIKLVSTSYMLEKSLSDPSTDFDRIH
STVKSPRSELKHEKSWRRQKISGTRERKAATTLGLILGAFVICWLPFFVKELVVNVCEKCKIS
EEMSNFLAWLGYLNSLINPLIYTIFNEDFKKAFQKLVRCRY

>MmHtr2a-NP_766400.1
MEILCEDNISLSSIPNSLMQLGDDSRLYPNDFNSRDANTSEASNWTIDAENRTNLSCEGYLPP
TCLSILHLQEKNWSALLTTVVIILTIAGNILVIMAVSLEKKLQNATNYFLMSLAIADMLLGFLVMP
VSMLTILYGYRWPLPSKLCAVWIYLDVLFSTASIMHLCAISLDRYVAIQNPIHHSRFNSRTKAF
LKIIAVWTISVGISMPIPVFGLQDDSKVFKEGSCLLADDNFVLIGSFVAFFIPLTIMVITYFLTIKSL
QKEATLCVSDLSTRAKLSSFSFLPQSSLSSEKLFQRSIHREPGSYAGRRTMQSISNEQKACK
VLGIVFFLFVVMWCPFFITNIMAVICKESCNENVIGALLNVFVWIGYLSSAVNPLVYTLFNKTYR
SAFSRYIQCQYKENRKPLQLILVNTIPTLAYKSSQLQVGQKKNSQEDAEPTANDCSMVTLGN
QHSEEMCTDNIETVNEKVSCV

>MmHtr2b-NP_032337.2
MASSYKMSEQSTTSEHILQKTCDHLILTNRSGLETDSVAEEMKQTVEGQGHTVHWAALLILA
VIIPTIGGNILVILAVALEKRLQYATNYFLMSLAIADLLVGLFVMPIALLTIMFEAIWPLPLALCPA
WLFLDVLFSTASIMHLCAISLDRYIAIKKPIQANQCNSRATAFIKITVVWLISIGIAIPVPIKGIETD
VINPHNVTCELTKDRFGSFMVFGSLAAFFAPLTIMVVTYFLTIHTLQKKAYLVKNKPPQRLTR
WTVPTVFLREDSSFSSPEKVAMLDGSHRDKILPNSSDETLMRRMSSVGKRSAQTISNEQRA
SKALGVVFFLFLLMWCPFFITNLTLALCDSCNQTTLKTLLEIFVWIGYVSSGVNPLIYTLFNKTF
REAFGRYITCNYRATKSVKALRKFSSTLCFGNSMVENSKFFTKHGIRNGINPAMYQSPMRLR
SSTIQSSSIILLDTLLTENDGDKAEEQVSYI

>MmHtr2c-NP_032338.3
MVNLGTAVRSLLVHLIGLLVWQFDISISPVAAIVTDTFNSSDGGRLFQFPDGVQNWPALSIVVI
IIMTIGGNILVIMAVSMEKKLHNATNYFLMSLAIADMLVGLLVMPLSLLAILYDYVWPLPRYLCP
VWISLDVLFSTASIMHLCAISLDRYVAIRNPIEHSRFNSRTKAIMKIAIVWAISIGVSVPIPVIGLR
DESKVFVNNTTCVLNDPNFVLIGSFVAFFIPLTIMVITYFLTIYVLRRQTLMLLRGHTEEELRNIS
LNFLKCCCKKGDEEENAPNPNPDQKPRRKKKEKRPRGTMQAINNEKKASKVLGIVFFVFLIM
WCPFFITNILSVLCGKACNQKLMEKLLNVFVWIGYVCSGINPLVYTLFNKIYRRAFSKYLRCDY
KPDKKPPVRQIPRVAATALSGRELNVNIYRHTNERVVRKANDTEPGIEMQVENLELPVNPSN
VVSERISSV

>MmHtr4-NP_032339.2
MDKLDANVSSNEGFRSVEKVVLLTFLAVVILMAILGNLLVMVAVCRDRQLRKIKTNYFIVSLAF
ADLLVSVLVMPFGAIELVQDIWAYGEMFCLVRTSLDVLLTTASIFHLCCISLDRYYAICCQPLV
YRNKMTPLRIALMLGGCWVLPMFISFLPIMQGWNNIGIVDVIEKRKFSHNSNSTWCVFMVNK
PYAITCSVVAFYIPFLLMVLAYYRIYVTAKEHAQQIQMLQRAGATSESRPQPADQHSTHRMRT

ETKAAKTLCVIMGCFCFCWAPFFVTNIVDPFIDYTVPEQVWTAFLWLGYINSGLNPFLYAFLN
KSFRRAFLIILCCDDERYKRPPILGQTVPCSTTTINGSTHVLRDTVECGGQWESRCHLTATSP
LVAAQPSDT

>MmHtr5a-NP_032340.2
MDLPVNLTSFSLSTPSSLEPNRSLDTEVLRPSRPFLSAFRVLVLTLLGFLAAATFTWNLLVLAT
ILKVRTFHRVPHNLVASMAISDVLVAVLVMPLSLVHELSGRRWQLGRRLCQLWIACDVLCCT
ASIWNVTAIALDRYWSITRHLEYTLRTRKRVSNVMILLTWALSTVISLAPLLFGWGETYSEPSE
ECQVSREPSYTVFSTVGAFYLPLCVVLFVYWKIYRAAKFRMGSRKTNSVSPVPEAVEVKNAT
QHPQMVFTVRHATVTFQTEGDTWREQKEQRAALMVGILIGVFVLCWFPFFVTELISPLCSW
DVPAIWKSIFLWLGYSNSFFNPLIYTAFNRSYSSAFKVFFSKQQ

>MmHtr5b-P31387.1
MEVSNLSGATPGLAFPPGPESCSDSPSSGRSMGSTPGGLILPGREPPFSAFTVLVVTLLVLLI
AATFLWNLLVLVTILRVRAFHRVPHNLVASTAVSDVLVAVLVMPLSLVSELSAGRRWQLGRS
LCHVWISFDVLCCTASIWNVAAIALDRYWTITRHLQYTLRTRSRASALMIAITWALSALIALAPL
LFGWGEAYDARLQRCQVSQEPSYAVFSTCGAFYLPLAVVLFVYWKIYKAAKFRFGRRRRAV
VPLPATTQAKEAPPESEMVFTARRRATVTFQTSGDSWREQKEKRAAMMVGILIGVFVLCWIP
FFLTELISPLCACSLPPIWKSIFLWLGYSNSFFNPLIYTAFNKNYNNAFKSLFTKQR

>MmHtr6-NP_001364025.1
MVPEPGPVNSSTPAWGPGPPPAPGGSGWVAAALCVVIVLTAAANSLLIALICTQPALRNTSN
FFLVSLFTSDLMVGLVVMPPAMLNALYGRWVLARGLCLLWTAFDVMCCSASILNLCLISLDR
YLLILSPLRYKLRMTAPRALALILGAWSLAALASFLPLLLGWHELGKARTSAPGQCRLLASLPY
VLVASGVTFFLPSGAICFTYCRILLAARKQAVQVASLTTGTATAGQALETLQVPRTPRPGMES
ADSRRLTTKHSRKALKASLTLGILLSMFFVTWLPFFVASIAQAVCDCISPGLFDVLTWLGYCN
STMNPIIYPLFMRDFKRALGRFVPCVHCPPEHRASPASPSMWTSHSGARPGLSLQQVLPLP
LPPNSDSDSASGGTSGLQLTAQLLLPGEATRDPPPPTRAPTVVNFFVTDSVEPEIRQHPLGS
PMN

>MmHtr7-NP_032341.2
MMDVNSSGRPDLYGHLRSLILPEVGRRLQDLSPDGGAHSVVSSWMPHLLSGFPEVTASPA
PTWDAPPDNVSGCGEQINYGRVEKVVIGSILTLITLLTIAGNCLVVISVCFVKKLRQPSNYLIVS
LALADLSVAVAVMPFVSVTDLIGGKWIFGHFFCNVFIAMDVMCCTASIMTLCVISIDRYLGITR
PLTYPVRQNGKCMAKMILSVWLLSASITLPPLFGWAQNVNDDKVCLISQDFGYTIYSTAVAFY
IPMSVMLFMYYQIYKAARKSAAKHKFSGFPRVQPESVISLNGVVKLQKEVEECANLSRLLKH
ERKNISIFKREQKAATTLGIIVGAFTVCWLPFFLLSTARPFICGTSCSCIPLWVERTCLWLGYA
NSLINPFIYAFFNRDLRTTYRSLLQCQYRNINRKLSAAGMHEALKLAERPERSEFVLQNCDHC
GKKGHDT

>MmDrd1-NP_001278730.1
MAPNTSTMDETGLPVERDFSFRILTACFLSLLILSTLLGNTLVCAAVIRFRHLRSKVTNFFVISL
AVSDLLVAVLVMPWKAVAEIAGFWPFGSFCNIWVAFDIMCSTASILNLCVISVDRYWAISSPF
QYERKMTPKAAFILISVAWTLSVLISFIPVQLSWHKAKPTWPLDGNFTSLEDAEDDNCDTRLS
RTYAISSSLISFYIPVAIMIVTYTSIYRIAQKQIRRISALERAAVHAKNCQTTTGNGNPVECSQSE
SSFKMSFKRETKVLKTLSVIMGVFVCCWLPFFISNCMVPFCGSEETQPFCIDSITFDVFVWFG
WANSSLNPIIYAFNADFQKAFSTLLGCYRLCPTTNNAIETVSINNNGAVMFSSHHEPRGSISK
DCNLVYLIPHAVGSSEDLKREEAGGIPKPLEKLSPALSVILDYDTDVSLEKIQPVTHSGQHST

>MmDrd2-NP_034207.2
MDPLNLSWYDDDLERQNWSRPFNGSEGKPDRPHYNYYAMLLTLLIFIIVFGNVLVCMAVSRE
KALQTTTNYLIVSLAVADLLVATLVMPWVVYLEVVGEWKFSRIHCDIFVTLDVMMCTASILNLC
AISIDRYTAVAMPMLYNTRYSSKRRVTVMIAIVWVLSFTISCPLLFGLNNTDQNECIIANPAFVV
YSSIVSFYVPFIVTLLVYIKIYIVLRKRRKRVNTKRSSRAFRANLKTPLKGNCTHPEDMKLCTVI
MKSNGSFPVNRRRMDAARRAQELEMEMLSSTSPPERTRYSPIPPSHHQLTLPDPSHHGLH
SNPDSPAKPEKNGHAKIVNPRIAKFFEIQTMPNGKTRTSLKTMSRRKLSQQKEKKATQMLAIV
LGVFIICWLPFFITHILNIHCDCNIPPVLYSAFTWLGYVNSAVNPIIYTTFNIEFRKAFMKILHC

>MmDrd3-NP_031903.1
MAPLSQISSHINSTCGAENSTGVNRARPHAYYALSYCALILAIIFGNGLVCAAVLRERALQTTT
NYLVVSLAVADLLVATLVMPWVVYLEVTGGVWNFSRICCDVFVTLDVMMCTASILNLCAISID
RYTAVVMPVHYQHGTGQSSCRRVALMITAVWVLAFAVSCPLLFGFNTTGDPSICSISNPDFVI
YSSVVSFYVPFGVTVLVYARIYMVLRQRRRKRILTRQNSQCISIRPGFPQQSSCLRLHPIRQF
SIRARFLSDATGQMEHIEDKPYPQKCQDPLLSHLQPLSPGQTHGELKRYYSICQDTALRHPN
FEGGGGMSQVERTRNSLSPTMAPKLSLEVRKLSNGRLSTSLKLGPLQPRGVPLREKKATQM
VVIVLGAFIVCWLPFFLTHVLNTHCQACHVSPELYRATTWLGYVNSALNPVIYTTFNIEFRKAF
LKILSC

>MmDrd4-NP_031904.1
MGNSSATEDGGLLAGRGPESLGTGAGLGGAGAAALVGGVLLIGLVLAGNSLVCVSVASERT
LQTPTNYFIVSLAAADLLLAVLVLPLFVYSEVQGGVWLLSPRLCDTLMAMDVMLCTASIFNLC
AISVDRFVAVTVPLRYNQQGQCQLLLIAATWLLSAAVASPVVCGLNDVPGRDPAVCCLENRD
YVVYSSVCSFFLPCPLMLLLYWATFRGLRRWEAARHTKLHSRAPRRPSGPGPPVSDPTQG
PFFPDCPPPLPSLRTSPSDSSRPESELSQRPCSPGCLLADAALPQPPEPSSRRRRGAKITGR
ERKAMRVLPVVVGAFLVCWTPFFVVHITRALCPACFVSPRLVSAVTWLGYVNSALNPIIYTIF
NAEFRSVFRKTLRLRC

>MmDrd5-NP_038531.1

MLPPGRNGTAHRARLGLQRQLAQVDAPGGSAAPLGPAQVVTAGLLTLLIVWTLLGNVLVCA
AIVRSRHLRAKMTNIFIVSLAVSDLFVALLVMPWKAVAEVAGYWPFGAFCDIWVAFDIMCSTA
SILNLCIISVDRYWAISRPFRYERKMTQRVALVMVALAWTLSILISFIPVQLNWHRDKAGSQGR
EGLLSNETPWEEGWELDGRTENCDSSLNRTYAISSSLISFYIPVAIMIVTYTRIYRIAQVQIRRI
SSLERAAEHAQSCRSRGACEPDPSLRASIKKETKVFKTLSVIMGVFVCCWLPFFILNCMVPF
CSSGDAQGPRTGFPCVSETTFDIFVWFGWANSSLNPIIYAFNADFRKVFAQLLGCSHLCFRT
PVQTVNISNELISYNQDTVFHREIAAAYVHMIPNAVSSGDREVGEEEEAEEEGPFDHMSQISP
TTPDGDLAAESVWELDCEEEVSLGKISPLTPNCFHKTA

>MmHrh1-NP_001239571.1
MSLPNTSSASEDKMCEGNRTAMASPQLLPLVVVLSSISLVTVGLNLLVLYAVRSERKLHTVG
NLYIVSLSVADLIVGAVVMPMNILYLIMTKWSLGRPLCLFWLSMDYVASTASIFSVFILCIDRYR
SVQQPLRYLRYRTKTRASATILGAWFLSFLWVIPILGWHHFTPLAPELREDKCETDFYNVTW
FKIMTAIINFYLPTLLMLWFYVKIYKAVRRHCQHRQLTNGSLPTFLEIKLRSEDAKEGAKKPGK
ESPWGVQKRPSRDPTGGLDQKSTSEDPKVTSPTVFSQEGERETVTRPCFRLDVMQTQPVP
EGDARGSKANDQTLSQPKMDEQSLSTCRRISETSEDQTLVDRQSFSRTTDSDTSIEPGLGK
VKARSRSNSGLDYIKVTWKRLRSHSRQYVSGLHLNRERKAAKQLGCIMAAFILCWIPYFIFFM
VIAFCNSCCSEPVHMFTIWLGYINSTLNPLIYPLCNENFKKTFKKILHIRS

>MmHrh2-NP_001010973.1
MEPNGTVHSCCLDSIALKVTISVVLTTLIFITVAGNVVVCLAVSLNRRLRSLTNCFIVSLAATDL
LLGLLVMPFSAIYQLSFKWSFGQVFCNIYTSLDVMLCTASILNLFMISLDRYCAVTDPLRYPVL
VTPVRVAISLVFIWVISITLSFLSIHLGWNSRNGTRGGNDTFKCKVQVNEVYGLVDGMVTFYL
PLLIMCVTYYRIFKIAREQAKRINHISSWKAATIREHKATVTLAAVMGAFIVCWFPYFTAFVYR
GLRGDDAVNEVVEGIVLWLGYANSALNPILYATLNRDFRMAYQQLFHCKLASHNSHKTSLRL
NNSLLSRSQSREGRWQEEKPLKLQVWSGTELTHPQGSPVRTRLSHSSCLLSLSLLSFIWKL
GTWIHHRRPFQPSLHISA

>MmHrh3-NP_598610.1
MERAPPDGLMNASGALAGEAAAAGGARGFSAAWTAVLAALMALLIVATVLGNALVMLAFVA
DSSLRTQNNFFLLNLAISDFLVGAFCIPLYVPYVLTGRWTFGRGLCKLWLVVDYLLCASSVFN
IVLISYDRFLSVTRAVSYRAQQGDTRRAVRKMALVWVLAFLLYGPAILSWEYLSGGSSIPEGH
CYAEFFYNWYFLITASTLEFFTPFLSVTFFNLSIYLNIQRRTRLRLDGGREAGPEPPPDAQPSP
PPAPPSCWGCWPKGHGEAMPLHRYGVGEAGPGVETGEAGLGGGSGGGAAASPTSSSGS
SSRGTERPRSLKRGSKPSASSASLEKRMKMVSQSITQRFRLSRDKKVAKSLAIIVSIFGLCWA
PYTLLMIIRAACHGHCVPDYWYETSFWLLWANSAVNPVLYPLCHYSFRRAFTKLLCPQKLKV
QPHGSLEQCWK

>MmHrh4-NP_694727.1

MSESNSTGILPPAAQVPLAFLMSSFAFAIMVGNAVVILAFVVDRNLRHRSNYFFLNLAISDFLV
GLISIPLYIPHVLFNWNFGSGICMFWLITDYLLCTASVYNIVLISYDRYQSVSNAVSYRAQHTGI
MKIVAQMVAVWILAFLVNGPMILASDSWKNSTNTKDCEPGFVTEWYILTITMLLEFLLPVISVA
YFNVQIYWSLWKRRALSRCPSHAGFSTTSSSASGHLHRAGVACRTSNPGLKESAASRHSES
PRRKSSILVSLRTHMNSSITAFKVGSFWRSESAALRQREYAELLRGRKLARSLAILLSAFAIC
WAPYCLFTIVLSTYPRTERPKSVWYSIAFWLQWFNSFVNPFLYPLCHRRFQKAFWKILCVTK
QPALSQNQSVSS

>MmAdra1a-NP_001258689.1
MVLLSENASEGSNCTHPPAQVNISKAILLGVILGGLIIFGVLGNILVILSVACHRHLHSVTHYYIV
NLAVADLLLTSTVLPFSAIFEILGYWAFGRVFCNIWAAVDVLCCTASIMGLCIISIDRYIGVSYPL
RYPTIVTQRRGVRALLCVWALSLVISIGPLFGWRQQAPEDETICQINEEPGYVLFSALGSFYV
PLTIILVMYCRVYVVAKRESRGLKSGLKTDKSDSEQVTLRIHRKNVPAEGSGVSSAKNKTHFS
VRLLKFSREKKAAKTLGIVVGCFVLCWLPFFLVMPIGSFFPNFKPPETVFKIVFWLGYLNSCIN
PIIYPCSSQEFKKAFQNVLRIQCLRRRQSSKHALGYTLHPPSQAVEGQHRGMVRIPVGSGET
FYKISKTDGVREWKFFSSMPQGSARITMPKDQSACTTARVRSKSFLQVCCCVGSSTPRPEE
NHQVPTIKIHTISLGENGEEV

>MmAdra1b-P97717.2
MNPDLDTGHNTSAPAHWGELKDANFTGPNQTSSNSTLPQLDVTRAISVGCLGAFILFAIVGNI
LVILSVACNRHLRTPTNYFIVNLAIADLLLSFTDLPFSATLEVLGYWVLGRIFCDIWAAVDVLCC
TASILSLCAISIDRYIGVRYSLQYPTLVTRRKAILALLSVWVLSTVISIGPLLGWKEPAPNDDKE
CGVTEEPFYALFSSLGSFYIPLAVILVMYCRVYIVAKRTTKNLEAGVMKEMSNSKELTLRIHSK
NFHEDTLSSTKAKGHNPRSSIAVKLFKFSREKKAAKTLGIVVGMFILCWLPFFIALPLGSLFST
LKPPDAVFKVVFWLGYFNSCLNPIIYPCSSKEFKRAFMRILGCQCRGGRRRRRRRRLGACA
YTYRPWTRGGSLERSQSRKDSLDDSGSCMSGSQRTLPSASPSPGYLGRGTQPPVELCAFP
EWKPGALLSLPEPPGRRGRLDSGPLFTFKLLGEPESPGTEGDASNGGCDTTTDLANGQPG
FKSNMPLAPGHF

>MmAdra1d-P97714.1
MTFRDILSVTFEGPRASSSTGGSGAGGGAGTVGPEGPAVGGVPGATGGSAVVGTGSGED
NQSSTAEAGAAASGEVNGSAAVGGLVVSAQGVGVGVFLAAFILTAVAGNLLVILSVACNRHL
QTVTNYFIVNLAVADLLLSAAVLPFSATMEVLGFWPFGRTFCDVWAAVDVLCCTASILSLCTI
SVDRYVGVRHSLKYPAIMTERKAAAILALLWAVALVVSVGPLLGWKEPVPPDERFCGITEEV
GYAIFSSVCSFYLPMAVIVVMYCRVYVVARSTTRSLEAGIKREPGKASEVVLRIHCRGAATSA
KGNPGTQSSKGHTLRSSLSVRLLKFSREKKAAKTLAIVVGVFVLCWFPFFFVLPLGSLFPQLK
PSEGVFKVIFWLGYFNSCVNPLIYPCSSREFKRAFLRLLRCQCRRRRRRLWPSLRPPLASLD
RRPALRLCPQPAHRTPRGSPSPHCTPRPGLRRHAGGAGFGLRPSKASLRLREWRLLGPLQ
RPTTQLRAKVSSLSHKFRSGGARRAETACALRSEVEAVSLNVPQDGAEAVICQAYEPGDLS

NLRETDI

>MmAdra2a-NP_031443.4
MGSLQPDAGNSSWNGTEAPGGGTRATPYSLQVTLTLVCLAGLLMLFTVFGNVLVIIAVFTSR
ALKAPQNLFLVSLASADILVATLVIPFSLANEVMGYWYFGKVWCEIYLALDVLFCTSSIVHLCAI
SLDRYWSITQAIEYNLKRTPRRIKAIIVTVWVISAVISFPPLISIEKKGAGGGQQPAEPSCKIND
QKWYVISSSIGSFFAPCLIMILVYVRIYQIAKRRTRVPPSRRGPDACSAPPGGADRRPNGLGP
ERGAGPTGAEAEPLPTQLNGAPGEPAPAGPRDGDALDLEESSSSEHAERPPGPRRPDRGP
RAKGKTRASQVKPGDSLPRRGPGAAGPGASGSGHGEERGGGAKASRWRGRQNREKRFT
FVLAVVIGVFVVCWFPFFFTYTLIAVGCPVPSQLFNFFFWFGYCNSSLNPVIYTIFNHDFRRAF
KKILCRGDRKRIV

>MmAdra2b-NP_033763.3
MVHQEPYSVQATAAIASAITFLILFTIFGNALVILAVLTSRSLRAPQNLFLVSLAAADILVATLIIPF
SLANELLGYWYFWRAWCEVYLALDVLFCTSSIVHLCAISLDRYWAVSRALEYNSKRTPRRIK
CIILTVWLIAAVISLPPLIYKGDQRPEPHGLPQCELNQEAWYILASSIGSFFAPCLIMILVYLRIYV
IAKRSHCRGLGAKRGSGEGESKKPRPGPAAGGVPASAKVPTLVSPLSSVGEANGHPKPPR
EKEEGETPEDPEARALPPNWSALPRSVQDQKKGTSGATAEKGAEEDEEEVEECEPQTLPA
SPASVFNPPLQQPQTSRVLATLRGQVLLSKNVGVASGQWWRRRTQLSREKRFTFVLAVVIG
VFVVCWFPFFFSYSLGAICPQHCKVPHGLFQFFFWIGYCNSSLNPVIYTIFNQDFRRAFRRIL
CRQWTQTGW

>MmAdra2c-NP_031444.2
MASPALAAALAAAAAEGPNGSDAGEWGSGGGANASGTDWVPPPGQYSAGAVAGLAAVVG
FLIVFTVVGNVLVVIAVLTSRALRAPQNLFLVSLASADILVATLVMPFSLANELMAYWYFGQV
WCGVYLALDVLFCTSSIVHLCAISLDRYWSVTQAVEYNLKRTPRRVKATIVAVWLISAVISFPP
LVSFYRRPDGAAYPQCGLNDETWYILSSCIGSFFAPCLIMGLVYARIYRVAKLRTRTLSEKRG
PAGPDGASPTTENGLGKAAGENGHCAPPRTEVEPDESSAAERRRRRGALRRGGRRREGA
EGDTGSADGPGPGLAAEQGARTASRSPGPGGRLSRASSRSVEFFLSRRRRARSSVCRRKV
AQAREKRFTFVLAVVMGVFVLCWFPFFFSYSLYGICREACQLPEPLFKFFFWIGYCNSSLNP
VIYTVFNQDFRRSFKHILFRRRRRGFRQ

>MmAdrb1-NP_031445.2
MGAGALALGASEPCNLSSAAPLPDGAATAARLLVLASPPASLLPPASEGSAPLSQQWTAGM
GLLLALIVLLIVVGNVLVIVAIAKTPRLQTLTNLFIMSLASADLVMGLLVVPFGATIVVWGRWEY
GSFFCELWTSVDVLCVTASIETLCVIALDRYLAITSPFRYQSLLTRARARALVCTVWAISALVS
FLPILMHWWRAESDEARRCYNDPKCCDFVTNRAYAIASSVVSFYVPLCIMAFVYLRVFREAQ
KQVKKIDSCERRFLGGPARPPSPEPSPSPGPPRPADSLANGRSSKRRPSRLVALREQKALK
TLGIIMGVFTLCWLPFFLANVVKAFHRDLVPDRLFVFFNWLGYANSAFNPIIYCRSPDFRKAF

QRLLCCARRAACRRRAAHGDRPRASGCLARAGPPPSPGAPSDDDDDDAGTTPPARLLEP
WTGCNGGTTTVDSDSSLDEPGRQGFSSESKV

>MmAdrb2-NP_031446.2
MGPHGNDSDFLLAPNGSRAPDHDVTQERDEAWVVGMAILMSVIVLAIVFGNVLVITAIAKFER
LQTVTNYFIISLACADLVMGLAVVPFGASHILMKMWNFGNFWCEFWTSIDVLCVTASIETLCVI
AVDRYVAITSPFKYQSLLTKNKARVVILMVWIVSGLTSFLPIQMHWYRATHKKAIDCYTEETC
CDFFTNQAYAIASSIVSFYVPLVVMVFVYSRVFQVAKRQLQKIDKSEGRFHAQNLSQVEQDG
RSGHGLRRSSKFCLKEHKALKTLGIIMGTFTLCWLPFFIVNIVHVIRDNLIPKEVYILLNWLGYV
NSAFNPLIYCRSPDFRIAFQELLCLRRSSSKTYGNGYSSNSNGRTDYTGEPNTCQLGQERE
QELLCEDPPGMEGFVNCQGTVPSLSVDSQGRNCSTNDSPL

>MmAdrb3-NP_038490.2
MAPWPHRNGSLALWSDAPTLDPSAANTSGLPGVPWAAALAGALLALATVGGNLLVIIAIART
PRLQTITNVFVTSLAAADLVVGLLVMPPGATLALTGHWPLGETGCELWTSVDVLCVTASIETL
CALAVDRYLAVTNPLRYGTLVTKRRARAAVVLVWIVSAAVSFAPIMSQWWRVGADAEAQEC
HSNPRCCSFASNMPYALLSSSVSFYLPLLVMLFVYARVFVVAKRQRHLLRRELGRFSPEESP
PSPSRSPSPATGGTPAAPDGVPPCGRRPARLLPLREHRALRTLGLIMGIFSLCWLPFFLANV
LRALAGPSLVPSGVFIALNWLGYANSAFNPVIYCRSPDFRDAFRRLLCSYGGRGPEEPRAVT
FPASPVEARQSPPLNRFDGYEGARPFPT

>MmChrm1-NP_001106167.1
MNTSVPPAVSPNITVLAPGKGPWQVAFIGITTGLLSLATVTGNLLVLISFKVNTELKTVNNYFL
LSLACADLIIGTFSMNLYTTYLLMGHWALGTLACDLWLALDYVASNASVMNLLLISFDRYFSV
TRPLSYRAKRTPRRAALMIGLAWLVSFVLWAPAILFWQYLVGERTVLAGQCYIQFLSQPIITF
GTAMAAFYLPVTVMCTLYWRIYRETENRARELAALQGSETPGKGGGSSSSSERSQPGAEG
SPESPPGRCCRCCRAPRLLQAYSWKEEEEEDEGSMESLTSSEGEEPGSEVVIKMPMVDPE
AQAPTKQPPKSSPNTVKRPTKKGRDRGGKGQKPRGKEQLAKRKTFSLVKEKKAARTLSAIL
LAFILTWTPYNIMVLVSTFCKDCVPETLWELGYWLCYVNSTVNPMCYALCNKAFRDTFRLLLL
CRWDKRRWRKIPKRPGSVHRTPSRQC

>MmChrm2-NP_987076.2
MNNSTNSSNNGLAITSPYKTFEVVFIVLVAGSLSLVTIIGNILVMVSIKVNRHLQTVNNYFLFSL
ACADLIIGVFSMNLYTLYTVIGYWPLGPVVCDLWLALDYVVSNASVMNLLIISFDRYFCVTKPL
TYPVKRTTKMAGMMIAAAWVLSFILWAPAILFWQFIVGVRTVEDGECYIQFFSNAAVTFGTAI
AAFYLPVIIMTVLYWHISRASKSRIKKEKKEPVANQDPVSPSLVQGRIVKPNNNNMPGGDGG
LEHNKIQNGKAPRDGGTENCVQGEEKESSNDSTSVSAVASNMRDDEITQDENTVSTSLGHS
KDDNSRQTCIKIVTKTQKGDACTPTSTTVELVGSSGQNGDEKQNIVARKIVKMTKQPAKKKP
PPSREKKVTRTILAILLAFIITWAPYNVMVLINTFCAPCIPNTVWTIGYWLCYINSTINPACYALC

NATFKKTFKHLLMCHYKNIGATR

>MmChrm3-NP_150372.1
MTLHSNSTTSPLFPNISSSWVHSPSEAGLPLGTVSQLDSYNISQTSGNFSSNDTSSDPLGGH
TIWQVVFIAFLTGFLALVTIIGNILVIVAFKVNKQLKTVNNYFLLSLACADLIIGVISMNLFTTYIIM
NRWALGNLACDLWLSIDYVASNASVMNLLVISFDRYFSITRPLTYRAKRTTKRAGVMIGLAW
VISFVLWAPAILFWQYFVGKRTVPPGECFIQFLSEPTITFGTAIAAFYMPVTIMTILYWRIYKET
EKRTKELAGLQASGTEAEAENFVHPTGSSRSCSSYELQQQGTKRSSRRKYGGCHFWFTTK
SWKPSAEQMDQDHSSSDSWNNNDAAASLENSASSDEEDIGSETRAIYSIVLKLPGHSTILNS
TKLPSSDNLQVPDKDLGTMDVERNAHKLQAQKSMDDRDNCQKDFSKLPIQLESAVDTAKTS
DTNSSVDKTTAALPLSFKEATLAKRFALKTRSQITKRKRMSLIKEKKAAQTLSAILLAFIITWTP
YNIMVLVNTFCDSCIPKTYWNLGYWLCYINSTVNPVCYALCNKTFRTTFKMLLLCQCDKRKR
RKQQYQQRQSVIFHKRVPEQAL

>MmChrm4-XP_006498717.1
MANFTPVNGSSANQSVRLVTTAHNHLETVEMVFIATVTGSLSLVTVVGNILVMLSIKVNRQLQ
TVNNYFLFSLACADLIIGAFSMNLYTLYIIKGYWPLGAVVCDLWLALDYVVSNASVMNLLIISFD
RYFCVTKPLTYPARRTTKMAGLMIAAAWVLSFVLWAPAILFWQFVVGKRTVPDNQCFIQFLS
NPAVTFGTAIAAFYLPVVIMTVLYIHISLASRSRVHKHRPEGPKEKKAKTLAFLKSPLMKPSIKK
PPPGGASREELRNGKLEEAPPPALPPPPRPVADKDTSNESSSGSATQNTKERPPTELSTTE
AATTPALPAPTLQPRTLNPASKWSKIQIVTKQTGSECVTAIEIVPATPAGMRPAANVARKFASI
ARNQVRKKRQMAARERKVTRTIFAILLAFILTWTPYNVMVLVNTFCQSCIPERVWSIGYWLCY
VNSTINPACYALCNATFKKTFRHLLLCQYRNIGTAR

>MmChrm5-NP_991352.2
MEGESYHNETTVNGTPVNHQALERHGLWEVITIAAVTAVVSLMTIVGNVLVMISFKVNSQLKT
VNNYYLLSLACADLIIGIFSMNLYTTYILMGRWVLGSLACDLWLALDYVASNASVMNLLVISFD
RYFSITRPLTYRAKRTPKRAGIMIGLAWLVSFILWAPAILCWQYLVGKRTVPPDECQIQFLSEP
TITFGTAIAAFYIPVSVMTILYCRIYRETEKRTKDLADLQGSDSVAEVKKRKPAHRTLLRSFFSC
PRPSLAQRVRNQASWSSSRRSTSTTGKPTQATDLSADWEKAEQVTNCSSCPSSEDEAKAT
TDPVFQVVCKNEAKESPGKEFNTQETKETFVSPRTENNDYDTPKYFLSPGAAHRLKSQKCV
AYKFRLVVKADGTQETNNGCRKVKIMPCSFPVSKDPSTKGLDPHLSHQMTKRKRMVLVKER
KAAQTLSAILLAFIITWTPYNIMVLVSTFCDKCVPVTLWHLGYWLCYVNSTINPICYALCNRTF
RKTFKLLLLCRWKKKKVEEKLYWQGNSKLP
